# Predictors of Response to Web-Based Cognitive Behavioral Therapy With High-Intensity Face-to-Face Therapist Guidance for Depression: A Bayesian Analysis

**DOI:** 10.2196/jmir.4351

**Published:** 2015-09-02

**Authors:** Ragnhild Sørensen Høifødt, Matthias Mittner, Kjersti Lillevoll, Susanne Kvam Katla, Nils Kolstrup, Martin Eisemann, Oddgeir Friborg, Knut Waterloo

**Affiliations:** ^1^ Department of Psychology Faculty of Health Sciences UiT The Arctic University of Norway Tromsø Norway; ^2^ Division of Substance Abuse and Addiction Vestre Viken Hospital Trust Drammen Norway; ^3^ Department of Community Medicine, General Practice Research Unit Faculty of Health Sciences UiT The Arctic University of Norway Tromsø Norway

**Keywords:** treatment outcome, computer-assisted therapy, cognitive behavior therapy, depression, primary health care, Bayesian analysis

## Abstract

**Background:**

Several studies have demonstrated the effect of guided Internet-based cognitive behavioral therapy (ICBT) for depression. However, ICBT is not suitable for all depressed patients and there is a considerable level of nonresponse. Research on predictors and moderators of outcome in ICBT is inconclusive.

**Objective:**

This paper explored predictors of response to an intervention combining the Web-based program MoodGYM and face-to-face therapist guidance in a sample of primary care patients with mild to moderate depressive symptoms.

**Methods:**

Participants (N=106) aged between 18 and 65 years were recruited from primary care and randomly allocated to a treatment condition or to a delayed treatment condition. The intervention included the Norwegian version of the MoodGYM program, face-to-face guidance from a psychologist, and reminder emails. In this paper, data from the treatment phase of the 2 groups was merged to increase the sample size (n=82). Outcome was improvement in depressive symptoms during treatment as assessed with the Beck Depression Inventory-II (BDI-II). Predictors included demographic variables, severity variables (eg, number of depressive episodes and pretreatment depression and anxiety severity), cognitive variables (eg, dysfunctional thinking), module completion, and treatment expectancy and motivation. Using Bayesian analysis, predictors of response were explored with a latent-class approach and by analyzing whether predictors affected the slope of response.

**Results:**

A 2-class model distinguished well between responders (74%, 61/82) and nonresponders (26%, 21/82). Our results indicate that having had more depressive episodes, being married or cohabiting, and scoring higher on a measure of life satisfaction had high odds for positively affecting the probability of response. Higher levels of dysfunctional thinking had high odds for a negative effect on the probability of responding. Prediction of the slope of response yielded largely similar results. Bayes factors indicated substantial evidence that being married or cohabiting predicted a more positive treatment response. The effects of life satisfaction and number of depressive episodes were more uncertain. There was substantial evidence that several variables were unrelated to treatment response, including gender, age, and pretreatment symptoms of depression and anxiety.

**Conclusions:**

Treatment response to ICBT with face-to-face guidance may be comparable across varying levels of depressive severity and irrespective of the presence and severity of comorbid anxiety. Being married or cohabiting, reporting higher life satisfaction, and having had more depressive episodes may predict a more favorable response, whereas higher levels of dysfunctional thinking may be a predictor of poorer response. More studies exploring predictors and moderators of Internet-based treatments are needed to inform for whom this treatment is most effective.

**Trial Registration:**

Australian New Zealand Clinical Trials Registry number: ACTRN12610000257066; https://www.anzctr.org.au/trial_view.aspx?id=335255 (Archived by WebCite at http://www.webcitation.org/6GR48iZH4).

## Introduction

### Background

Several efficacious psychological and pharmacological treatments for depression exist [1]. A well-documented treatment is cognitive behavioral therapy (CBT), which has shown comparable effects as pharmacotherapy in treating mild to moderate depression with the additional benefit of reducing relapse [2,3].

The therapy model, structure, and short-term format of CBT make it highly suitable for delivery through self-help material. Delivery through Internet services is one example and several studies have demonstrated the efficacy of Internet-based CBT (ICBT) for depression, especially when guided by a therapist (eg, [4,5-8]). In fact, the treatment effects from guided ICBT and standard face-to-face treatment seem to be comparable [9-11]. Despite the positive results, ICBT is not suitable for all depressed patients because the problem of nonresponse is notable ranging from 50% to 65% (eg, [6,7,12]). Therefore, the question of which patients this treatment is effective for is important to address. The aim of this study is to examine pretreatment variables that can predict response to an ICBT protocol that was published previously [13].

### General Prognostic Factors

A number of studies have investigated factors predicting the course of depression in primary care and community settings. Factors associated with a poorer course of the depressive disorder include individual characteristics (eg, high levels of neuroticism [14,15]), socioeconomic factors (eg, low educational level [16,17], unemployment [16,17]), relational factors (eg, lack of social support [17-19], loneliness [17]), health-related variables (eg, somatic illness [17,18], severity of somatic symptoms [19,20], poor self-rated health [21], lower levels of mental [20] and global [15] functioning), and factors related to the depressive disorder (eg, baseline depressive severity [17-20,22], history of depression [23], duration of depressive episodes [16,18], dysthymia or double depression [15 24], and comorbidity with anxiety [17,19,24], substance abuse [22], or personality disorders [24]).

### Predictors of Response to Cognitive Behavioral Therapy

In the literature on treatment response, the concepts of prognostic and prescriptive factors are discussed [25]. The former represent nonspecific predictors of response and the latter represent moderators which refer to variables predicting differential treatment response between treatments [25,26]. The latter is most useful for informing which treatments seem most suitable for which patient characteristics or subpopulations [27].

Several patient characteristics have been suggested to influence response to CBT for depression. Patient expectancy, perceived treatment credibility, and improvement in the early phases of treatment seem to be powerful predictors of outcome in cognitive therapy and psychotherapy in general [28-32]. Demographic variables such as gender, age, education, and employment status are less consistently related to treatment outcomes [33-36]. However, in a recent study of treatment-resistant depression in primary care, age was found to moderate the effect of CBT with older patients gaining most benefit from this treatment [37]. In addition, married patients seem to respond consistently better to CBT compared to unmarried patients [38-40]. Many studies suggest poorer outcomes in terms of posttreatment symptoms for patients with high baseline depressive severity (eg, [41-43]). This relationship may depend on the definition of outcome; Van et al [34] propose that high initial severity may be associated with more difficulty achieving remission, whereas symptom change may be achieved more readily because higher severity leaves more room for improvement. In addition, regression to the mean effects can be expected to be stronger for those with a higher symptom load. Other features of the depressive disorder, such as high chronicity and younger age of onset, have been found to predict poorer response to CBT [29,38], but the predictive role of number of depressive episodes [29,30,39] and comorbid anxiety remains unclear [44-47]. With its relation to the proposed mechanism of change in CBT, the role of dysfunctional attitudes has received considerable attention and several studies conclude that high baseline levels of dysfunctional attitudes predict a poorer treatment response [29,33,39,48,49].

### Predictors of Response to Internet-Based Cognitive Behavioral Therapy

For ICBT, results concerning depressive severity are consistent with previous research on face-to-face CBT [4,50-56]. In contrast to previous research, studies of ICBT have found either no association between marital status and treatment response [6,52,57] or a positive association between being separated, widowed, or divorced and symptom reduction [53]. Two studies of younger and older adults, respectively, found more favorable outcomes for females [52,58]. Donker et al [59] found similar results in a sample with a broader age range, whereas others have not replicated this finding [4,6,12,56,57]. Age itself did not significantly predict outcome in these studies, with the exception of Donker et al [59] in which age was found to be a moderator because older individuals responded more favorably to CBT and younger individuals improved more with interpersonal therapy (IPT). Results have been mixed for educational level, employment status, dysfunctional attitudes, and for clinical variables such as number of depressive episodes and the presence of comorbidity (eg, [6,12,50-53,56,57,59]).

Treatment credibility refers to the extent to which patients endorse a treatment model as logical and meaningful, and 2 studies found this to be unrelated to outcomes of ICBT [60,61]. Results were mixed with respect to treatment expectancy [60,61]. One study indicated that although higher motivation was associated with greater adherence, low and moderate levels were related to better outcomes, perhaps due to unrealistic expectations and proneness to disappointment for highly motivated participants [57]. One may presume that greater adherence leads to better outcomes, but even on this point there are inconsistencies with some studies finding an association [5,60,62-65] and others not [12,54,57,66-68]. A review suggested that the impact of adherence may depend on how it is measured and that module completion may be more consistently related to outcomes for depression than measures such as number of log-ins [69].

### Aim of the Study

The aim of this study is to identify prognostic predictors of response to an intervention combining the Web-based program MoodGYM and high-intensity face-to-face therapist guidance in a sample of mildly to moderately depressed primary care patients. Data from a randomized controlled trial (RCT) comparing this intervention to a delayed treatment condition was used. Data from the treatment phase of the 2 groups were collapsed. This increased sample size in the treatment group, but also precludes a clear distinction between general prognostic factors and predictors specific to CBT. This limitation must be borne in mind when interpreting the results. Predictor variables were predominately chosen on the basis of previous research on CBT delivered face-to-face and over the Internet, but some measures were included for exploratory purposes.

Most patients with mild to moderate depression receive all their treatment in primary care where the availability of psychological treatments is often limited [70-73]. If implemented in primary care, this intervention could constitute an alternative to treatment as usual. This paper may indicate which patients in a depressed primary care population may benefit more or less from treatment with MoodGYM and therapist guidance.

Based on previous literature, we hypothesized that (1) more positive expectations would predict a more favorable response to treatment, (2) participants with higher baseline depression severity would improve more, and (3) a higher score on a measure of dysfunctional thinking would predict a poorer treatment response. Because the remaining predictor variables have yielded mixed results in previous studies, no specific hypotheses were formulated for these.

## Methods

### Study Design

The study was a RCT with 2 conditions: (1) a treatment condition comprising 6 weeks of Web-based CBT with face-to-face therapist guidance and (2) a 6-week waitlist for the same treatment during which time participants could also access treatment as usual. The research protocol was approved by the Regional Committee for Research Ethics in Northern Norway and the Human Ethics Committee of the Australian National University (ANU). The trial was registered in the Australian New Zealand Clinical Registry (ACTRN12610000257066). A more detailed account of the study methods is given in Høifødt et al [13].

### Participants and Procedure

Participants (N=106) were recruited from general practitioners (GPs), primary care nurses, and from waitlists of primary care referrals at 2 psychiatric outpatient clinics. Local GPs and primary care nurses were informed about the study and provided their patients with information about the project. Patients on waitlists at the psychiatric outpatient clinics at the Psychiatric Centre for Tromsø at the University Hospital of North Norway and at the Department of Psychology at UiT The Arctic University of Norway were invited by postal mail. Patients consented by signing an informed consent form. Consenting participants were screened for inclusion and randomly allocated to the 2 groups. The study inclusion criteria were (1) aged 18 to 65 years, (2) access to the Internet, and (3) a score between 10 and 40 on the Beck Depression Inventory-II (BDI-II), which indicates mild to moderately severe symptoms of depression. Individuals already attending CBT were excluded. Participants with suicidal intentions, concurrent psychosis, or alcohol or drug abuse disorders were excluded. Participants who used antidepressant medication were stabilized for 1 month prior to entering the trial.

Assessments and treatment took place at the Department of Psychology at UiT The Arctic University of Norway. Because the patients allocated to the 2 study arms showed comparable courses during treatment, data from the treatment phase of the 2 groups was combined to increase statistical power. Seven participants in the control group dropped out during the waiting period and did not complete the pretreatment assessments. Another 7 participants did not meet the inclusion criteria according to the BDI-II at the pretreatment assessment and were excluded, as were 7 participants who provided data only on 1 measurement occasion. In addition, 3 outliers with treatment duration exceeding far beyond that of the rest of the sample (>28 weeks) were excluded. Because slopes of BDI-II were modeled as a function of time, treatment duration is a critical variable; therefore, we chose to base our criterion for outliers on this scale.

### Intervention

The guided self-help intervention included (1) The Norwegian version of the ICBT program MoodGYM version 3 [74], (2) face-to-face therapist guidance of high-intensity, and (3) reminder emails between sessions.

The MoodGYM was originally developed at ANU as a free-of-charge automated Web intervention delivered to the public [75]. MoodGYM consists of 5 self-help modules and 29 exercises. The program is based on CBT and was developed to prevent and reduce symptoms of depression and anxiety among adolescents [76], but is efficacious for adult populations also [8,77-79]. MoodGYM focuses on identification and restructuring of dysfunctional thinking, activation of behavioral strategies to increase engagement in positive activities, as well as learning of stress reduction and problem-solving techniques.

Participants were introduced to the program and instructed to complete one module per week. After each module, participants received face-to-face support (15-30 minutes) from a psychologist (RSH or KL). The main elements of the sessions were reinforcement of progress, discussion of key messages from the modules, and helping participants to relate to the material and employ techniques from the program in their daily life. The full intervention included 8 sessions. The mean number of sessions attended was 7.0 (SD 2.2). Due to delays, some participants attended more sessions (9 sessions: n=8; 10 sessions: n=3; 11 sessions: n=1). Mean session length in minutes (excluding screening) was 28.1 (SD 6.9, range 15.8-48.6). Therapists aimed to meet participants weekly. However, the interval between sessions and the number of sessions were allowed to vary to meet individual needs. Thus, treatment duration varied between participants (mean 9.6, SD 4.8, range 1-22) and there was no fixed posttreatment time-point.

### Outcome

Several outcome measures were analyzed in the trial focusing on the effect of the intervention [13]. In this paper, analyses are restricted to predicting response on the BDI-II. The BDI-II was administered to all participants at baseline (before randomization) and before every consultation during the intervention phase. The control group also completed an assessment before entering online treatment (pretreatment).

The BDI-II is a 21-item self-report measure of severity of depressive symptoms during the past 2 weeks [80]. Studies consistently support the BDI-II as a reliable, internally consistent, and valid scale for assessing depression [80-82]. In this study, internal consistency (Cronbach alpha) ranged from .79 to .97 and was generally greater than .90 for the measurement occasions T1 to T11 (baseline to session 11).

### Predictors of Outcome

#### Demographic Variables

The variables gender, age, marital status, and employment status were collected during the screening interview before randomization. Marital status and employment status were dichotomized as married/cohabiting versus not married/cohabiting and being employed versus not being employed, respectively.

#### Severity Variables

This group of variables included pretreatment measures of severity of depressive and anxious symptoms and quality of life, as well as depression and anxiety diagnosis, number of depressive episodes, and alcohol use measured at baseline. In addition, previous treatment was included as a dichotomous variable (1=yes, 0=no) indicating whether participants had previously received pharmacotherapy or psychological treatment for depression.

Severity of anxiety and depression symptoms pretreatment was assessed with The Hospital Anxiety and Depression Scale (HADS). This inventory has 2 subscales of 7 items each, measuring depression and anxiety, respectively, and is reliable and valid [83,84]. In this study, Cronbach alpha was .67 and .81 for the depression and anxiety subscales, respectively.

Another measure of anxiety severity was The Beck Anxiety Inventory (BAI) [85]. The inventory possesses robust internal consistency, reliability, and validity [86-88]. Cronbach alpha in the present study was .92.

The Mini-International Neuropsychiatric Interview (MINI) [89] was used to identify participants who fulfilled the criteria for a major depressive episode (MDE) or any anxiety disorder, and to determine the number of previous depressive episodes (0=no lifetime MDE; 1=single lifetime MDE; 2=2-4 lifetime MDEs; 3=≥5 lifetime MDEs).

Alcohol use was assessed with the Alcohol Use Disorders Identification Test (AUDIT) [90]. The instrument has favorable internal consistency, reliability, and criterion validity [90,91]. Participants with scores greater than 20 were excluded from the study. Cronbach alpha in this study was .81.

Health-related quality of life was assessed with the EuroQol 5-Dimension Self-Report Questionnaire (EQ-5D) [92]. Respondents mark their level of functioning for each of 5 dimensions (mobility, self-care, usual activities, pain/ discomfort, and anxiety/depression).

The Satisfaction With Life Scale (SWLS) measures global life satisfaction according to the individual’s own criteria [93]. The scale has sound psychometric properties [94,95]. Cronbach alpha in this study was .78.

#### Cognitive Variables

Dysfunctional thinking and self-efficacy were explored as potential predictors of response. Dysfunctional thinking patterns were measured with the Warpy Thoughts Quiz, which is part of the first module of MoodGYM [96]. The 42-item quiz covers 7 areas of dysfunctional thinking: the need for approval, love, to succeed, and to be perfect; expectations of rights; influence on others; and the view that happiness depends on external things. Items are rated from 1 (strongly agree) to 5 (strongly disagree). Higher scores indicate more dysfunctional thinking. Norms were based on a sample aged 20 to 32 years (N=153) [97], and the scale demonstrates good internal consistency (Cronbach alpha=.77-.84) [96]. A 20-item short form of the scale correlates strongly with the Automatic Thoughts Questionnaire (*r*=.51) and moderately (*r*=.39) with measures of depression and anxiety [98].

The General Self-Efficacy Scale (GSE) assesses broad and stable beliefs about one’s ability to deal with various demands and challenges [99]. The GSE has satisfactory reliability and construct and criterion cross-cultural validity [100-103]. Cronbach alpha in this study was .89.

#### Expectancy, Motivation, and Use

Expectancy, attitudes toward using an Internet-based program, and motivation were measured after introducing CBT and MoodGYM using questions developed for the purpose of this study:

1. To which degree do you expect that an Internet-based self-help program can be helpful for your depressive symptoms?
2. How is your attitude toward using an Internet-based self-help program?
3. How likely is it that you will use this Internet-based self-help program?

For the first 2 questions, 5-point Likert scales (1=very high expectations, 5=very low expectations; 1=very negative attitude, 5=very positive attitude) were used. Responses to the item on motivation (question 3) were given on an 11-point scale from 0% to 100%. User data on module completion was registered online and was denoted by a number between zero and 4, with zero indicating no use and 4 indicating completion of the module.

### Statistical Analyses Using Bayesian Statistics

#### Motivation for Using Bayesian Methods

Bayesian methods were used for data analyses instead of the more commonly used null-hypothesis significance testing (NHST) approach (for a general introduction to Bayesian methods, see [104,105]). In a Bayesian framework, we directly estimated the posterior probability distribution of the parameters taking data and model structure into account. Bayesian methods are suitable in the current setting for several reasons. First, the use of Bayesian hierarchical modeling allows the design of custom models that are appropriate for the data without relying on approximations as is necessary in NHST methods. Furthermore, Bayesian modeling is highly flexible because the posterior distribution can be readily transformed into easily interpretable quantities and the uncertainty inherent to the analysis is propagated and available at each level of analysis. As such, Bayesian analysis relies much less on point estimates and an arbitrary choice of significance levels. Indeed, the strong critique on *P* values (regarding, for example, their biasing impact on which results are trusted/reported and the problems with their interpretation [106,107]) emerging in many relevant scientific fields such as medicine [108] and psychology [106] has triggered the development of Bayesian methods in these fields (eg, [109,110]). Instead of reporting *P* values and relying on the problematic concept of statistical significance using an arbitrary significance level, Bayesian methods report the results of an analysis in terms of probabilities, odds ratios, and Bayes factors that give a more graded and readily interpretable summary of the conclusions supported by the data.

Odds ratios are ratios of probabilities or densities indicating the probability of one event occurring relative to another. Similarly, the Bayes factor quantifies how much more likely one hypothesis is with respect to another by dividing the posterior model odds by the prior model odds. Note that the Bayes factor integrates the probability over the complete parameter space and, therefore, automatically punishes overly complex models. Jeffreys [111] discussed how Bayes factors could be interpreted in terms of strength of evidence for and against a hypothesis (see Table 1) and it has been shown that Bayes factors are less prone to overestimating effects from psychological experiments compared to *P* values [112].

Using Bayes factors, Bayesian modeling may quantify the support for the null hypothesis and to what extent the null hypothesis (H_0_) is more likely than the alternative (H_1_). This is advantageous compared to traditional NHST-based tests which can only “not reject” the null hypothesis. This is a desirable feature when investigating the potential impact of predictor variables on treatment efficiency.

**Table 1:** Evidence categories for Bayes factors (BF_10_).^a^

Bayes factor	Interpretation
>100	Decisive evidence for H_1_
30-100	Very strong evidence for H_1_
10-30	Strong evidence for H_1_
3-10	Substantial evidence for H_1_
1-3	Anecdotal evidence for H_1_
1	No evidence
1/3-1	Anecdotal evidence for H_0_
1/10-1/3	Substantial evidence for H_0_
1/30-1/10	Strong evidence for H_0_
1/100-1/30	Very strong evidence for H_0_
<1/100	Decisive evidence for H_0_

^a^ Adapted from Wetzels et al [112]. BF_10_ is the odds for the alternative hypothesis (H_1_) divided by the odds for the null hypothesis (H_0_).

#### Statistical Models

Depression scores from BDI-II were acquired for each individual over several weeks of treatment. Because the intervention allowed a flexible session schedule there was resulting variation in measurement occasions; therefore, the effects of time from treatment could not be disentangled. Because participants could use the self-help program between sessions, we hypothesized that participants would continuously benefit from the treatment between sessions. Therefore, time (in weeks) was chosen as the repeating variable because this was considered to be the most correct representation of the data. We conducted a model selection procedure (for details see Multimedia Appendix 1) to find the most faithful representation of our data from among a linear, a quadratic, and an exponential model. Based on the results from this procedure, we modeled the BDI-II scores on the individual level as an exponential function of time and constrained the individual regression coefficients by a group-level distribution (hierarchical model). In Bayesian analysis, the specification of prior belief is essential. We specified a weakly informative prior such that the estimates were allowed to vary across a large number of parameter values while constraining them to be in a plausible range [105,113].

We implemented 2 complementary models, one for predicting probability of responding to treatment and another one for quantifying the strength of the response. The models were fit using Markov chain Monte Carlo (MCMC) algorithms implemented in the Just Another Gibbs Sampler (JAGS) software [114] and convergence was ensured by visual inspection and the Gelman-Rubin diagnostic [115]. We also conducted posterior predictive checks to ensure that the model fit the data well [105] (see Figures S1-S3 in Multimedia Appendix 1).

#### Predicting Probability of Response

Response to depression treatment varies substantially across individuals [27]. Latent-class approaches allow for the modeling of different growth trajectories across subgroups and captures this unobserved heterogeneity in trajectories by employing a categorical latent variable [116,117]. Class membership is initially unknown, but is inferred based on observed data resulting in identified classes of individuals with more similar response patterns within each group than between groups [116]. Thus, different classes of individuals may vary around different mean growth curves with potentially unique forms and parameter values. This can be advantageous compared to conventional growth modeling which assumes that all individuals are drawn from the same population and estimates the average growth curve for this population [118]. Furthermore, covariates can be included in the model to predict class membership and, in this way, individual characteristics predicting differential trajectories may be identified. Previous investigations have successfully employed latent-class methods to identify different distributions for groups of responders and nonresponders to treatment [32,119,120]. Therefore, we chose to fit a model that assumed 2 different distributions from which subject-level parameters could be drawn. Predictor variables were used as regressors on probability of class membership using a logit link function (for details see Multimedia Appendix 1) resulting in estimates β_i_ for each predictor. The resulting model effectively distinguished between responders and nonresponders (see Figure S4 in Multimedia Appendix 1).

#### Predicting the Strength of Response

In a next step, we aimed to explain variation in responsiveness by identifying variables that correlated with the slope of the response. This is an alternative way to look at prediction of response and it has the advantage of being more directly comparable to previous studies because latent-class approaches have not been widely used in the field. We modeled this situation by adding the subject-level covariates as linear predictors on the estimate of the first-level regression slope. Because changes of the slope parameter in the exponential model are not reflected linearly (a unit change on a low slope parameter has strong impact whereas the same change on a higher slope parameter has less impact), we relied on the quadratic model for this approach. This resulted in estimates α_i_ for the regression coefficient for each predictor.

## Results

### Sample Characteristics

**Figure 1:**
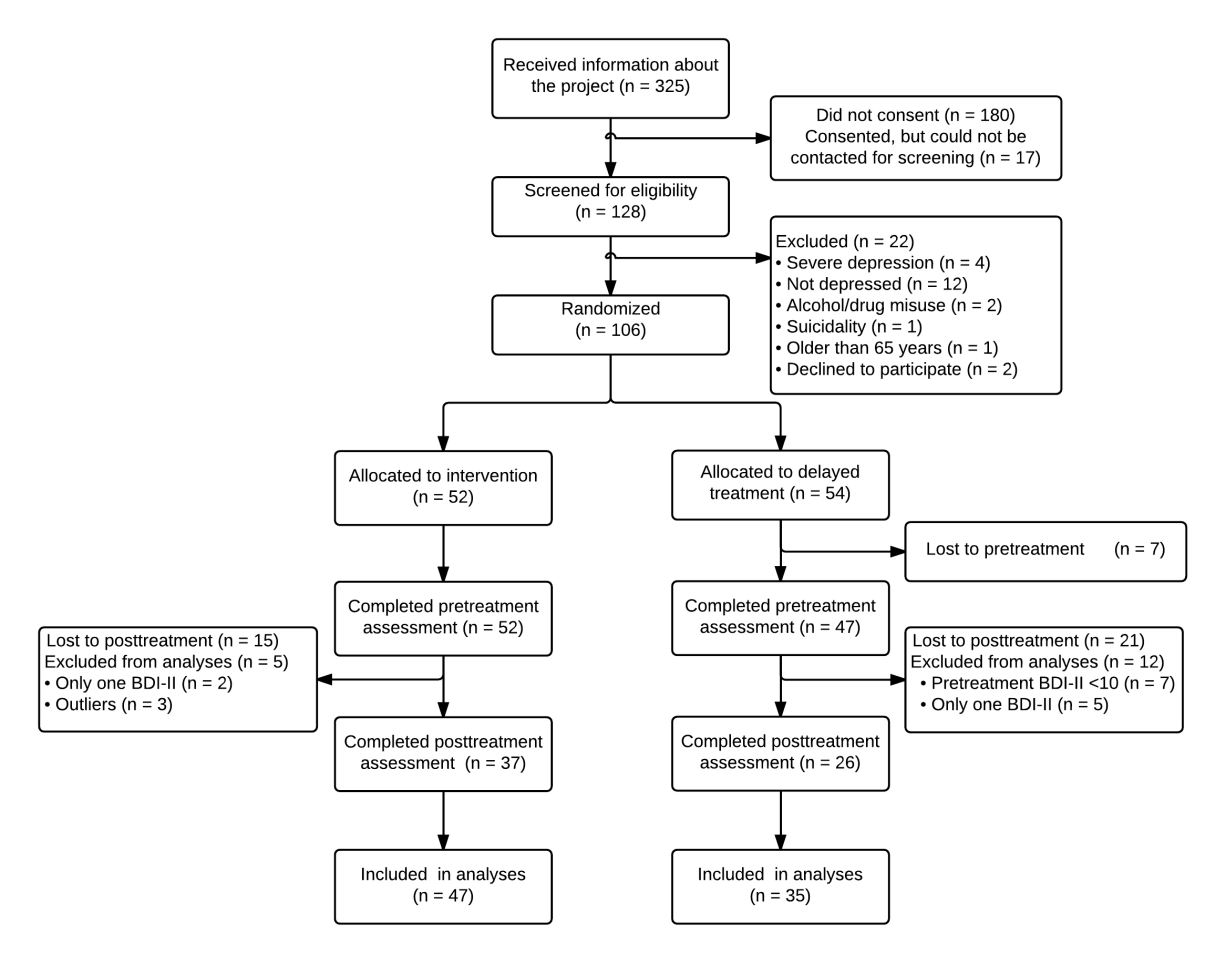
Flow of participants through the trial.

A total of 106 participants were included in the study and randomized to an intervention condition (n=52) or a delayed treatment control condition (n=54). Figure 1 describes the flow of participants through the trial. Of the 54 participants in the control group, 47 (87%) showed up for pretreatment assessment after being on a waitlist. For the control and intervention groups, 21 of 47 (45%) and 15 of 52 (29%) participants, respectively, dropped out between pre- and posttreatment assessments.

Treatment adherence was moderate with 31 of 52 participants (60%) in the intervention group and 20 of 54 participants (37%) in the control group adhering to treatment (completing MoodGYM and attending at least 7 sessions). The average number of completed modules and pretreatment characteristics of the sample are presented in Table 2. Distributions for the predictors are shown in Figure S5 in Multimedia Appendix 1.

**Table 2:** Participant characteristics (N=82).

Variables			Participants
**Demographic variables**			
	Gender (female), n (%)		60 (73)
	**Age (years)**		
		Mean (SD)	36.0 (11.7)
		Range	18-63
	Marital status (married/cohabiting), n (%)		44 (54)
	Educational level (higher education),^a^ n (%)		41 (50)
	Employment status (employed),^b^ n (%)		56 (68)
**Severity variables**			
	**Symptom measures,** ^c^ **mean (SD)**		
		Beck Depression Inventory-II	21.3 (6.6)
		Beck Anxiety Inventory	13.0 (10.2)
		HADS Depression	8.3 (2.9)
		HADS Anxiety	9.7 (4.1)
		Satisfaction With Life Scale	16.7 (5.1)
		EQ-5D	0.7 (0.2)
		AUDIT	5.0 (4.1)
	Depression diagnosis, n (%)		44 (54)
	**Number of major depressive episodes,** ^d^ **n (%)**		
		0	5 (6)
		1	27 (33)
		2-4	25 (31)
		≥5	19 (23)
	Comorbid anxiety,^e^ n (%)		27 (33)
	Earlier treatment,^f^ n (%)		49 (60)
	Present treatment (antidepressants or other^g^), n (%)		23 (28)
**Cognitive variables, mean (SD)**			
	Warpy Thoughts Quiz^f^		82.8 (25.1)
	General self-efficacy^f^		26.6 (4.9)
**Expectancy, motivation, and use**			
	Expectancy (1=very high expectations), mean (SD)		2.6 (0.7)
	Attitude (5=very positive), mean (SD)		4.1 (0.8)
	Motivation, mean (SD)		94.0 (12.2)
	Number of modules, mean (SD)		3.8 (1.7)
	**Treatment duration (weeks)**		
		Mean (SD)	9.6 (4.8)
		Range	1-22
	Treatment sessions, mean (SD)		7.0 (2.2)

^a^ Data for 1% (1/82) missing.

^b^ Employed: full-time or part-time employment. Not employed: unemployed, student, homemaker, long-term sick.

^c^ Hospital Anxiety and Depression Scale (HADS): 4% (3/82) missing, Satisfaction With Life Scale: 10% (8/82) missing, EuroQol 5-Dimension (EQ-5D) Self-Report Questionnaire: 11% (9/82) missing, Alcohol Use Disorders Identification Test (AUDIT): 1% (1/82) missing.

^d^ Data for 7% (6/82) missing.

^e^ Includes panic disorder, agoraphobia, social phobia, and generalized anxiety.

^f^ Data from 2% (2/82) missing.

^g^ Psychological therapy other than CBT.

### Predicting Probability of Response

**Figure 2:**
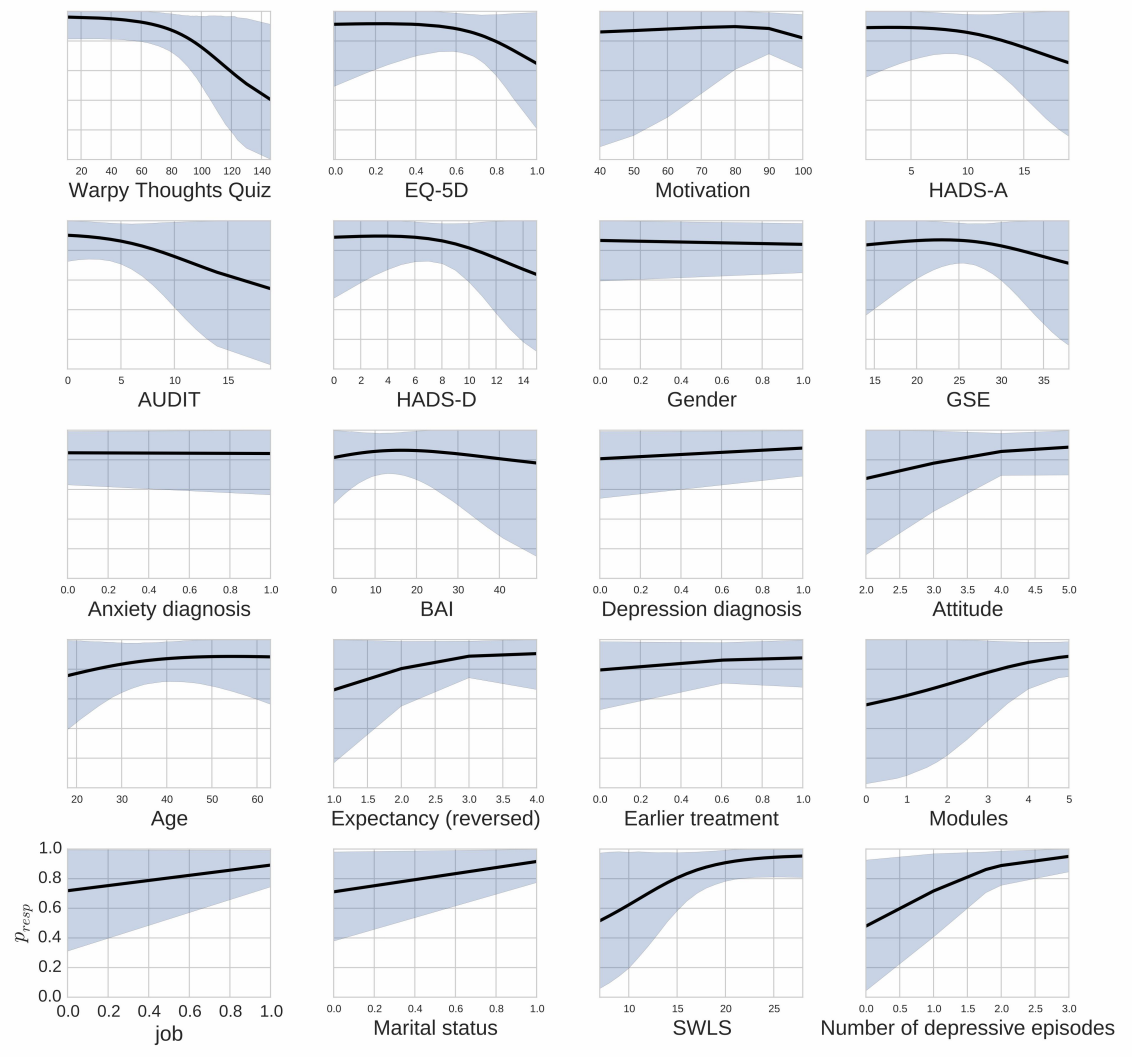
Probability of being in the responder group as a function of the predictor variables (assuming all other predictors remained at their baseline level). Black line is the mean posterior probability and shaded area is the 95% highest density interval.

The restricted 2-class model distinguished well between responders and nonresponders (ie, most participants either have a very low or a very high probability of belonging to the responder group, *P*_resp_) (see Figure S4 in Multimedia Appendix 1). Using *P*_resp_=.05 as split criterion, we found that 21 of 82 (26%) participants did not respond to treatment, whereas 61 of 82 (74%) did. These results were based on the conditional latent-class exponential model encompassing all predictor variables. A corresponding analysis using the quadratic model found qualitatively similar results. The results of the regression of the covariates on the probability to respond to the treatment are reported in Table 3. The odds ratios indicate the degree of evidence that each covariate has a positive/negative impact relative to the probability of the opposite (eg, as indicated in Table 3, it is almost 15 times more likely that a subject’s score on the Warpy Thoughts Quiz affects the probability of him or her responding to treatment negatively rather than positively). Thus, the odds ratios give an indication of the likely direction of the effect of a covariate on the probability of response, but do not delineate the strength of this effect. To give an indication of the strength of the effect, the probability of being in the responder group as a function of each of the covariates is plotted in Figure 2. This relatively complex reporting of the strength of effects was necessary for this analysis since the estimation of Bayes factors in latent-class models is computationally complicated and still a topic of ongoing research.

**Table 3:** Posterior mode, highest density interval (HDI), and odds ratios for the beta coefficients predicting probability of being a responder. The odds ratios indicate the probability that each covariate has a positive/negative impact relative to the probability of the opposite (+: positive effect; -: negative effect), but do not indicate the strength of this effect.

Variable^a^	Posterior mode (HDI)	OR |β_i_| >0
Warpy Thoughts Quiz	−0.93 (–2.27, 0.30)	14.55−
EQ-5D	−0.71 (–2.21, 0.76)	4.84−
Motivation	−0.70 (–2.13, 1.05)	3.42−
AUDIT	−0.49 (–1.82, 0.73)	3.67−
HADS-A	−0.48 (–1.87, 0.88)	3.11−
HADS-D	−0.44 (–1.72, 0.90)	2.99−
GSE	−0.21 (–1.71, 1.19)	1.70−
Gender	−0.15 (–1.46, 0.97)	1.74−
Anxiety diagnosis	0.04 (–1.29, 1.39)	1.10+
BAI	0.08 (–1.27, 1.63)	1.31+
Earlier treatment	0.33 (–0.88, 1.53)	2.44+
Depression diagnosis	0.33 (–0.96, 1.51)	2.19+
Age	0.37 (–0.86, 1.60)	2.56+
Expectancy (reversed)	0.39 (–0.77, 1.70)	3.23+
Attitude	0.42 (–0.87, 1.60)	2.54+
Modules	0.45 (–0.82, 1.82)	3.22+
Employment status	0.51 (–0.76, 1.81)	3.77+
Marital	0.83 (–0.48, 2.09)	8.17+
SWLS	0.88 (–0.44, 2.20)	10.92+
Number of depressive episodes	1.02 (–0.14, 2.28)	23.91+

^a^ AUDIT: Alcohol Use Disorders Identification Test; BAI: Beck Anxiety Inventory; EQ-5D: EuroQol 5-Dimension Self-Report Questionnaire; GSE: General Self-Efficacy Scale; HADS-A: Hospital Anxiety and Depression Scale-anxiety subscale; HADS-D: Hospital Anxiety and Depression Scale-depression subscale; SWLS: Satisfaction With Life Scale.

In summary, having had more depressive episodes, being married or cohabiting, and scoring higher on life satisfaction (SWLS) had high odds for positively affecting the probability of response. Tentative positive effects were found for the number of completed modules and having a paid job. Figure 2 shows that the effects are strongest for number of depressive episodes and scores on the SWLS with the probability for response approaching 1 for those with 5 or more depressive episodes and those with highest levels of life satisfaction, whereas those never having had a major depressive episode (only symptoms) and those with the lowest level of life satisfaction had only approximately .50 probability of response.

In the opposite direction, higher scores on the Warpy Thoughts Quiz were likely to have a negative effect on the probability of responding to treatment. Tentative negative effects were found for health-related quality of life (EQ-5D), motivation, expectancy, scores on both subscales of the HADS, and for alcohol use (AUDIT). Figure 2 shows that high scores on the Warpy Thoughts Quiz appear to be associated with a substantially reduced probability of response (*P*_resp_~.40). The impact of the other covariates are more limited (*P*_resp_~.60-.80 for participants with scores in the highest range; see Figure 2).

### Predicting the Strength of Response

The analysis of variation in responsiveness indicated that the predictors having the highest impact on response were largely consistent with the results from the latent-class model with the most important variables being the Warpy Thoughts Quiz, number of depressive episodes, life satisfaction (SWLS), module completion, and marital status. Results are summarized in terms of odds ratios in Table 4. Results from a separate analysis exploring the variation in responsiveness in the subgroup of responders (n=61) extracted by the latent-class model described in the previous section are presented in Multimedia Appendix 1.

Bayes factors quantify the strength of evidence for the null hypothesis (the covariate does not affect treatment response) and for the alternative hypothesis (the covariate affects response to treatment).

The results were largely consistent with the results from the odds ratio analyses with regard to which variables were most influential (see Table 5). However, the evidence was substantial only for the effect of marital status. There was substantial evidence for the null hypothesis for several variables, indicating that these variables are likely to be unrelated to treatment response in the present trial. This included the variables gender and age, and several severity variables including pretreatment symptoms of depression and anxiety, as well as treatment expectancy, attitude, and motivation. Inconsistent with the results from the odds ratio analyses, there was substantial evidence that the Warpy Thoughts Quiz was unrelated to treatment response.

**Table 4:** Posterior mode, highest density interval (HDI), and odds ratios for the α coefficients predicting the strength of the response. The α coefficients are the group-level regression coefficients on the slope of the treatment effect in the quadratic model (see Equation 5 in Multimedia Appendix 1). The odds ratios indicate the probability that each covariate has a positive/negative impact relative to the probability of the opposite (+: positive effect, –: negative effect), but do not indicate the strength of this effect.

Variable^a^	Posterior mode (HDI)	OR |α_i_| >0
Warpy Thoughts Quiz	−0.23 (–0.50, 0.05)	18.28−
Motivation	−0.20 (–0.50, 0.07)	13.00−
GSE	−0.17 (–0.45, 0.11)	7.35−
EQ-5D	−0.09 (–0.34, 0.16)	2.95−
Earlier treatment	−0.08 (–0.35, 0.19)	2.46−
AUDIT	−0.05 (–0.29, 0.18)	2.07−
HADS-D	−0.04 (–0.36, 0.25)	1.62−
Age	−0.04 (–0.31, 0.23)	1.53−
Attitude	−0.01 (–0.25, 0.23)	1.10−
BAI	−0.01 (–0.33, 0.30)	1.16−
Gender	0.01 (–0.21, 0.24)	1.29+
HADS-A	0.02 (–0.28, 0.33)	1.22+
Depression diagnosis	0.02 (–0.27, 0.30)	1.18+
Anxiety diagnosis	0.03 (–0.27, 0.33)	1.34+
Expectancy (reversed)	0.07 (–0.18, 0.32)	2.28+
Employment status	0.09 (–0.20, 0.34)	2.38+
Marital status	0.13 (–0.14, 0.41)	4.72+
Modules	0.18 (–0.12, 0.45)	7.43+
Number of depressive episodes	0.23 (–0.02, 0.49)	29.24+
SWLS	0.24 (–0.04, 0.52)	22.83+

^a^ AUDIT: Alcohol Use Disorders Identification Test; BAI: Beck Anxiety Inventory; EQ-5D: EuroQol 5-Dimension Self-Report Questionnaire; GSE: General Self-efficacy Scale; HADS-A: Hospital Anxiety and Depression Scale-anxiety subscale; HADS-D: Hospital Anxiety and Depression Scale-depression subscale; SWLS: Satisfaction With Life Scale.

**Table 5:** Bayes factors (BF_10_) quantifying the evidence for alternative hypotheses (H_1_) over the null hypothesis (H_0_). Variables are sorted with respect to its Bayes factor in ascending order. The null hypothesis is that the predictor does not have an impact on treatment response (H_0_: α_1_=0) and the alternative is that it does have an effect (H_1_: α_1_≠0). BF_10_ is the odds for H_1_ divided by the odds for H_0_.

Variable^a^	BF_10_	Evidence for
Earlier treatment	0.15	H_0_: substantial
Gender	0.15	H_0_: substantial
GSE	0.16	H_0_: substantial
BAI	0.16	H_0_: substantial
Expectancy	0.17	H_0_: substantial
Depression diagnosis	0.17	H_0_: substantial
EQ-5D	0.18	H_0_: substantial
Anxiety diagnosis	0.18	H_0_: substantial
HADS-A	0.20	H_0_: substantial
HADS-D	0.20	H_0_: substantial
Attitude	0.22	H_0_: substantial
Motivation	0.23	H_0_: substantial
Age	0.26	H_0_: substantial
Warpy Thoughts Quiz	0.29	H_0_: substantial
AUDIT	0.37	H_0_: anecdotal
Employment status	0.42	H_0_: anecdotal
Modules	0.47	H_0_: anecdotal
Number of depressive episodes	0.82	H_0_: anecdotal
SWLS	1.82	H_1_: anecdotal
Marital status	3.24	H_1_: substantial

^a^ AUDIT: Alcohol Use Disorders Identification Test; BAI: Beck Anxiety Inventory; EQ-5D: EuroQol 5-Dimension Self-Report Questionnaire; GSE: General Self-efficacy Scale; HADS-A: Hospital Anxiety and Depression Scale-anxiety subscale; HADS-D=Hospital Anxiety and Depression Scale-depression subscale; SWLS: Satisfaction With Life Scale.

## Discussion

### Principal Findings

This paper explored predictors to a treatment combining the MoodGYM program and high-intensity face-to-face guidance. Using Bayesian methods and a latent-class approach, a 2-class model classifying 74% of participants as responders and 26% as nonresponders was identified. The variation in responsiveness was also explored by analyzing whether predictors affected the slope of response. The results suggest that treatment effects were unrelated to baseline depressive severity, gender, and age. In addition, the presence and severity of comorbid anxiety did not predict differential response to treatment. Having a partner and reporting higher life satisfaction at baseline were associated with a more favorable treatment response. Results also indicated that having experienced more depressive episodes may predict more positive treatment effects, whereas higher scores on the Warpy Thoughts Quiz, which is a measure of dysfunctional thinking, may predict poorer response to treatment.

### Limitations

The results of this study must be interpreted in light of some methodological limitations. Despite merging data from both treatment groups, the size of the sample is limited and may be too small to allow reliable testing of effects. Small sample sizes are a common problem in research on prediction of treatment outcome [121]. Collapsing the data from the 2 groups increased the sample size in the treatment group, but precluded the identification of predictors or moderators of differential treatment response [27]. This means that this study cannot accurately distinguish between nonspecific predictors of good prognosis, nonspecific predictors of response to any treatment, and moderators (predicts differential response to treatments). In an effort to ameliorate this limitation, we separately investigated whether the individual predictors could explain the change in the BDI-II score during the waiting time for the waitlist control group. Due to the limited sample size, Bayes factors indicated no evidence for the alternative hypothesis for any of the predictors and could neither establish confidence in the null hypothesis for most of the variables.

Multiple comparisons in small samples also introduces a risk of chance findings. Studies with low power have a high chance of overestimating effect sizes or even making sign errors (eg, [122]). Bayesian methods allow us to model all data in a joint context and reduce the multiple comparison problem by constraining individual model coefficients by an overarching distribution (for details see [104]). In addition, formulating the results in terms of probabilities and odds ratios rather than making dichotomized decisions about whether or not a variable serves as a predictor or not can prevent overinterpretation of results.

Another limitation is that the intervention allowed a flexible session schedule and hence a variation in the spacing between measurement occasions. This means that the effects of time from treatment cannot be disentangled. Because participants could use the self-help program between sessions, we hypothesized that participants would continuously benefit from the treatment also between sessions. Therefore, time was chosen as the repeating variable because this was considered to be the most correct representation of the data.

The choice of outcome and predictor variables may also be criticized. Although demographic variables and baseline axis-I diagnoses were well covered, several variables that may have important contributions, such as personality variables, were not investigated. Therefore, these results can only give a partial description of factors influencing treatment response. The sole reliance on self-report is another limitation. Furthermore, treatment expectancy, attitudes, and motivation were measured using invalidated single items developed for this study. In addition, the convergent and discriminant validity of the Warpy Thoughts Quiz have not been established. This leaves uncertainty regarding how well these constructs were captured and calls for caution in interpreting the results.

A limitation of the 2-class model was that we were unable to estimate Bayes factors due to statistical complexity. This would have provided additional information about the strength of effects. Bayesian methods are a field of active research and development, and improved methods will surely be available in the future.

Finally, although a strength of the study is the recruitment of a relatively heterogeneous sample of primary care patients with regard to the range of depression and anxiety symptoms, the generalizability of the results is uncertain because the sample was a self-selected group. Nevertheless, an estimated uptake of 39% indicates that the trial sample may be representative of a considerable proportion of the targeted patient group [13]. Because some participants were excluded from analyses (eg, participants present at only one measurement occasion), results are based on a subsample of trial participants which further limits generalizability.

### Variables Unrelated to Treatment Response

Bayesian methods may be used to indicate the likelihood of the null hypotheses. The present analyses provide substantial evidence for the absence of any effect for several variables, such as pretreatment symptoms of depression and anxiety, depression or anxiety diagnosis, earlier treatment, and the demographic variables gender and age. This implies that MoodGYM combined with face-to-face guidance of relatively high intensity may be expected to work equally well for adult patients of varying ages, for women and men, and for various mild to moderate depressive symptom profiles, as well as for patients with comorbid anxiety of varying severity. Previous results regarding the predictive role of anxiety have been mixed [44-47,51,56,59,123]. With regard to depressive severity, several studies of CBT have found a larger response in terms of symptoms change for patients with higher severity (eg, [34,43,50,52,55]). However, these patients also tend to have more difficulties with achieving remission [39,43,124]. This trial did not find evidence for more improvement among participants having higher initial depressive severity; nevertheless, the results suggest that patients with higher depressive severity appear to benefit from treatment. Whether remission was achieved at comparable rates for participants with more or less severe depression cannot be answered by the present analyses. In addition, because the range of symptom severities was restricted because patients with severe depression were excluded and the proportion of patients having severe anxiety was small, no conclusions can be drawn with regard to more severe cases.

### Predictors of Improved Response

Being married or cohabiting was the most robust predictor of favorable response to treatment. This effect was evident both in the latent-class model and the analysis exploring the strength of response, and the Bayes factor indicated substantial evidence for a predictive effect. These results are in accordance with previous research on CBT delivered face-to-face [38-40]. In fact, some studies have suggested that marital status may be a prescriptive predictor for better outcomes in CBT compared to medications or IPT [38,40]. Although, this study cannot identify moderators, these past results indicate that having a partner is likely to be a predictor of treatment response and not merely of good prognosis. Supportive relationships were emphasized in interviews with participants from the current trial [125]. Participants described how important others encouraged them and facilitated their engagement in treatment (eg, by helping them make time to use MoodGYM or attend sessions). This strengthened their hope for recovery and motivation. Although important others also include friends and other family, one may hypothesize that living with a partner may facilitate such reinforcing processes. Also, being married or cohabiting may reflect a better ability to establish and maintain close relationships and this may in itself be an important factor for success in treatments that include interaction with a therapist [39]. This study included high-intensity face-to-face support. This may explain why this effect was evident in the current trial, whereas most studies of ICBT have failed to find any relation between marital status and response [6,52,57]. Replications within other contexts may decide whether this effect is unique to interventions including face-to-face contact or if similar processes operate also in Internet-based interventions including less support.

Life satisfaction also emerged as a possible predictor of better response to treatment, although the Bayes factor analysis indicated only anecdotal evidence for this effect. Life satisfaction may be regarded as an indirect measure of illness severity. The SWLS does not directly tap into constructs such as affect, but it is significantly negatively correlated with measures of depression and anxiety [94,95]. This result is consistent with an early study of ICBT in which higher quality of life, although assessed with a different scale, was associated with better outcomes [51]. However, this has not been replicated in other studies [56,59]. Why health-related quality of life (EQ-5D) showed tendencies toward predicting more inferior response in this study is more of a riddle. However, the 2 scales assess quite different constructs with the SWLS focusing on how satisfied individuals are with life according to their own criteria and not based on the presence or absence of specific ailments or impairments. The EQ-5D, on the other hand, focuses on the latter. These 2 constructs need not be highly correlated as is supported in this study’s data (*r*=.23). Whether life satisfaction is a more potent predictor of better treatment response remains to be replicated.

The results indicate that more depressive episodes have high odds for predicting a more favorable response. However, the result of the Bayes factor analysis was more ambiguous. This result is puzzling given that high rates of recurrence have been related to poor treatment outcomes [30,51] and treatment resistance [126,127] in previous studies. However, the findings are inconsistent and other studies have found no negative effect of high rates of recurrence on treatment outcomes [4,38,39,53,59]. There are some possible explanations for this finding. Compared with participants with a single or no depressive episodes, more participants with recurrent depression received antidepressant medication or additional psychological therapy. Although most did not receive additional treatment (~65%) and medications were stabilized for 1 month before entering the trial, one cannot rule out the possibility of this influencing the treatment effect. This would be consistent with a meta-analysis finding significantly better effects when adding psychotherapy to pharmacotherapy [128]. Another explanation may be related to the nature of recurrent depression in the general population because studies have suggested that subsequent episodes are shorter in duration than first episodes and have a mean duration of only 3 months [129,130]. This sample was recruited from GPs and is likely to be more similar to a general population sample than to a clinical population recruited from specialist mental health services. In accordance with these epidemiological studies, recurrent depression may be a predictor of shorter episode duration in general population samples. Finally, given that this finding was not fully robust across analyses and was in the opposite direction of most previous results, it may represent a chance finding as a result of random fluctuations in small samples.

The effect of module completion was ambiguous with the odds ratios indicating a tentative positive effect, but the Bayes factor indicating anecdotal support for no effect. Previous results have been mixed on the association between adherence to treatment and response [56,57,63-65,67-69]. The addition of supportive sessions in this trial may have confounded the effect of module completion and although there was high correlation between completing modules and attending sessions (*r*=.86), a measure reflecting adherence to both treatment components could have been a more potent predictor.

### Predictors of Poorer Response

The negative predictive effect of high scores on the Warpy Thoughts Quiz was evident in both models, but was not supported by the Bayes factor, which challenges the robustness of the finding. The Warpy Thoughts Quiz has not been used previously in studies of prediction. It is not entirely equivalent to the much-used Dysfunctional Attitude Scale [131], but taps into many of the same constructs including perfectionism and the need for success, love, and approval [96,132]. Worse treatment response has been associated with higher levels of dysfunctional attitudes in previous studies of face-to-face CBT [29,39,48,49] and some studies of ICBT [59], but not others [50,56]. Dysfunctional attitudes moderated treatment response in one study in which those with severe dysfunctional attitudes responded better to IPT and those with lower levels experienced better effects with CBT [29]. Again, this can indicate that this variable may be a predictor of response to treatment rather than a predictor of general prognosis. A proposed explanation is that patients having less severe dysfunctional attitudes may have greater cognitive flexibility [39] making them more able to profit from utilizing cognitive techniques [29].

### The Role of Expectations and Motivation

The results were somewhat mixed for treatment expectancy, attitude, and motivation with some analyses indicating a possible negative effect of motivation and expectancy, whereas the Bayes factors indicated substantial support for no effect for all 3 variables. The lack of effects in this study is inconsistent with our hypothesis and with previous studies of face-to-face therapy in which expectancy is considered an important predictor of outcome [29,31]. However, results have been inconclusive with respect to ICBT [60,61]. These results may be due to the fact that most individuals entering a research trial have fairly positive attitudes, expectations, and high motivation, which restricts the range of these variables as is reflected by the distributions displayed in Figure S5 in Multimedia Appendix 1. These variables may be more valuable predictors in a regular practice setting. In addition, these constructs were assessed using single items, which call the validity of these measures into question.

### Conclusion

The findings of the present study indicate that within a population of primary care patients with mild to moderate depression, treatment response to Web-based CBT with face-to-face guidance of high intensity was comparable across varying levels of initial depressive severity and irrespective of the presence and severity of comorbid anxiety. Whether the treatment is suitable for more severe depression is still uncertain. Treatment effects were also comparable for men and women and for patients of various ages. Being married or cohabiting and reporting higher life satisfaction predicted more favorable response to treatment. More positive response was also indicated for individuals with more previous depressive episodes, whereas having a higher level of dysfunctional thinking may predict poorer treatment response.

The purpose of this paper was primarily exploratory. Therefore, the results must be interpreted as hypotheses to inform further research rather than firm conclusions. Nevertheless, the results add to the knowledge base concerning differential treatment response, knowledge that is crucial for further implementation of Internet-based treatments in regular practice. Future studies should continue to explore predictors and, preferably, moderators of different Internet-based treatments compared to face-to-face treatments. In addition, studies exploring different patterns of response may also give important information about the differential response of various subgroups of patients.

## Appendix

Multimedia Appendix 1 Supplemental material: Details on statistical methods and additional analyses.

Multimedia Appendix 2 CONSORT-EHEALTH checklist V1.6.2 [133].

## Abbreviations

ANU: Australian National University
AUDIT: Alcohol Use Disorders Identification Test
BAI: Beck Anxiety Inventory
BDI-II: Beck Depression Inventory-II
BF: Bayes factor
CBT: cognitive behavioral therapy
EQ-5D: EuroQol 5-Dimension Self-Report Questionnaire
GP: general practitioner
GSE: General Self-efficacy Scale
HADS-A: Hospital Anxiety and Depression Scale-Anxiety subscale
HADS-D: Hospital Anxiety and Depression Scale-Depression subscale
HDI: highest density interval
ICBT: Internet-based cognitive behavioral therapy
IPT: interpersonal therapy
MCMC: Markov chain Monte Carlo
MDE: major depressive episode
NHST: null-hypothesis significance testing
Presp: probability of response
RCT: randomized controlled trial
SWLS: Satisfaction With Life Scale
