# Supplementary material for: Predictors of Response to Web-Based Cognitive Behavioral Therapy With High-Intensity Face-to-Face Therapist Guidance for Depression: A Bayesian Analysis
Source: J Med Internet Res. 2015 Sep 2;17(9):e197. doi: 10.2196/jmir.4351 (PMC4642793; doi:10.2196/jmir.4351)
Supplement: Multimedia Appendix 1 [file jmir_v17i9e197_app1.pdf]

Multimedia Appendix 1: Supplemental material

The following sections give a detailed account of the specification and selection of the model and the statistical analysis. In addition, in the results and discussion sections additional descriptive statistics and a supplemental analysis investigating predictors of the strength of response within the responder group is reported and discussed.

Methods

Statistical analysis

Model specification. Depression scores from BDI-II were acquired for each individual i over several weeks of treatment. As the intervention allowed a flexible session schedule, and hence the variation in measurement occasions, the effects of time from treatment cannot be disentangled. Because participants could use the self-help program between sessions, we hypothesized that participants would continuously benefit from the treatment also between sessions. Time was therefore chosen as the repeating variable as this was considered to be the most correct representation of the data. We modelled the BDI-II scores as being normally distributed with individual mean and standard deviation μ_i and σ_i

$$\text{BDI-II}_i \text{ (week)} \sim N(\mu_i, \sigma_i).$$

The individual mean is a function of time (weeks of treatment)

$$\mu_i = f(\text{week}; \theta)$$

with individual parameters θ and function f. We used three different functional forms for f (linear, quadratic and exponential) and compared them in terms of model-selection criteria:

$$f_{\text{lin}}(\text{week}) = a_i + b_i\,\text{week} \quad (1)$$

$$f_{\text{quad}}(\text{week}) = a_i + b_i\,\text{week} + c_i\,\text{week}^2 \quad (2)$$

$$f_{\text{exp}}(\text{week}) = a_i \exp(-b_i\,\text{week}) \quad (3)$$

The standard-deviation was modeled by a group-level gamma-distribution

$$\sigma_i \sim \text{Gamma}(r_\sigma, \lambda_\sigma)$$

with $r_\sigma = \frac{m_\sigma^2}{d_\sigma^2}$ and $\lambda_\sigma = \frac{m_\sigma}{d_\sigma^2}$ where m_σ is the mean and d_σ the standard deviation of the gamma-distribution.

The individual parameters (depending on the choice of f) are modeled by group-level distributions

$$\begin{aligned} c_i &\sim N(\mu_c, \sigma_c) \\ b_i &\sim N(\mu_b, \sigma_b) \\ a_i &\sim N(\mu_a, \sigma_a). \end{aligned}$$

Prior distribution. We assigned weakly informative priors to the group-level parameters such that the estimates were allowed to vary across a large number of parameter values while constraining them to be in a plausible range [1, 2]. The results were robust to the choice of prior. During model fitting, we experimented with different “degrees of non-informativeness” by changing the standard-deviation and limits of the prior distributions, but results remained unchanged. Concretely, the reported analyses used the following prior

$$\begin{aligned} m_\sigma &\sim \text{Gamma}(1,.25) \\ d_\sigma &\sim \text{Gamma}(1,.5) \end{aligned}$$

following a recommendation by Kruschke[3]. In addition, the mean for the first-level intercepts a_i received a non-informative prior across the range of allowed values

$$\mu_a \sim \text{Uniform}(0,60).$$

The hyperparameters for the coefficients b_i and c_i followed the same prior distributions

$$\mu_x \sim N(0,50)$$

for $x \in \{b, c\}$ and the group-level standard deviation for all three coefficients followed

$$\sigma_x \sim \text{Uniform}(0,50)$$

($x \in \{a, b, c\}$).

Model-fitting. We sampled from the posterior distribution of the parameters given the model using markov-chain monte-carlo (MCMC) algorithms implemented in the JAGS software [4]. All fits used 3 parallel chains, each with a burn-in period of 5000 samples and an adaptation period of 500 samples. Chains were initialized at single-subject maximum-likelihood fits and we sampled 100000 samples from the converged chains. We used a thinning factor of 2, indicating that we dropped every second sample from the MCMC chains to reduce autocorrelation in the series. Resulting samples were visually inspected for convergence to ensure good mixing behavior. We also applied the Gelman-Rubin diagnostic [5] and ensured that all reported results had $\hat{R} \leq 1.05$ indicating good convergence.

Model-selection. For model selection we used the Deviance information criterion [DIC; 6] which is a generalization of Akaikes information criterion (AIC) to hierarchical models (see Table S1). Differences in DIC larger than 10 can be considered strong [7].

Table S1. Model specification and selection criteria for linear, quadratic and exponential models.

Model number	Name	Specification	DIC[a]
1	Linear	f(week) = $a_i + b_i$ week	2962
2	Quadratic	f(week) = $a_i + b_i$ week + c_i week^2	2935
3	Exponential	f(week) = a_i exp(-b_i week)	2901

[a]DIC = Deviance information criterion.

Posterior predictive checks. In order to ensure that the model fit the data well, we conducted posterior predictive checks. In accordance with the recommendations of Gelman et

al. [1], we conducted graphical posterior predictive checks by plotting individual data and model fit. Figure S1 displays the posterior predictive plots for the exponential model. Posterior predictive plots for the linear and quadratic model are displayed in Figure S2 and S3, respectively.

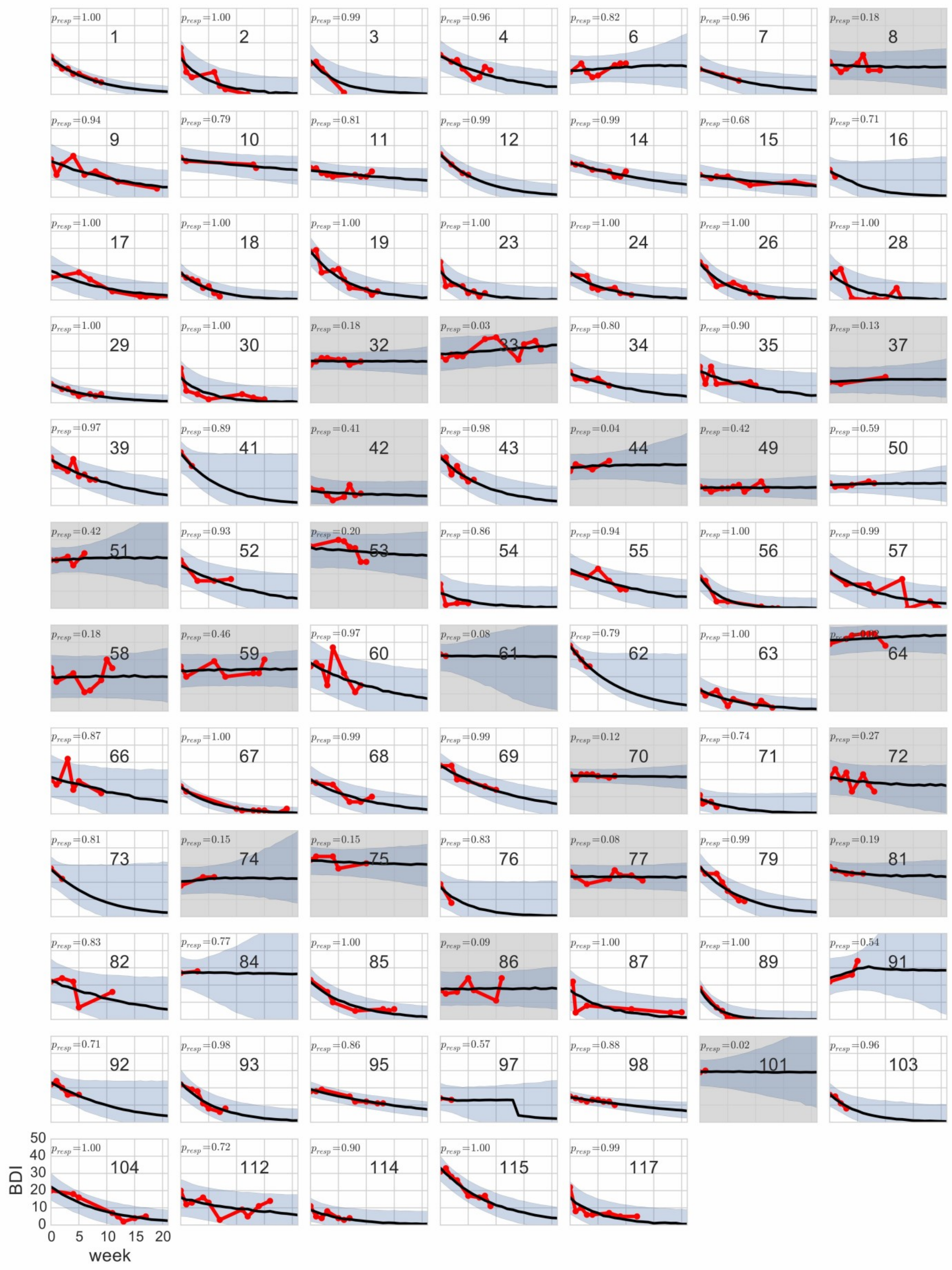

Figure S1. Posterior predictive plots for the exponential model. Each plot is data from a single subject over time (in weeks). Red solid line is data, black line is the mode of the posterior distribution estimated from the Markov-chain Monte-Carlo samples, and the shaded area indicates the 95 % highest density interval (HDI). Grey plots are individuals categorized as non-responders ($p_{resp} < .5$). The value for p_{resp} in the top left corner of each plot gives the estimated probability that each subject responded to the treatment. Note the rare occurrence of borderline cases (e.g., patient 50).

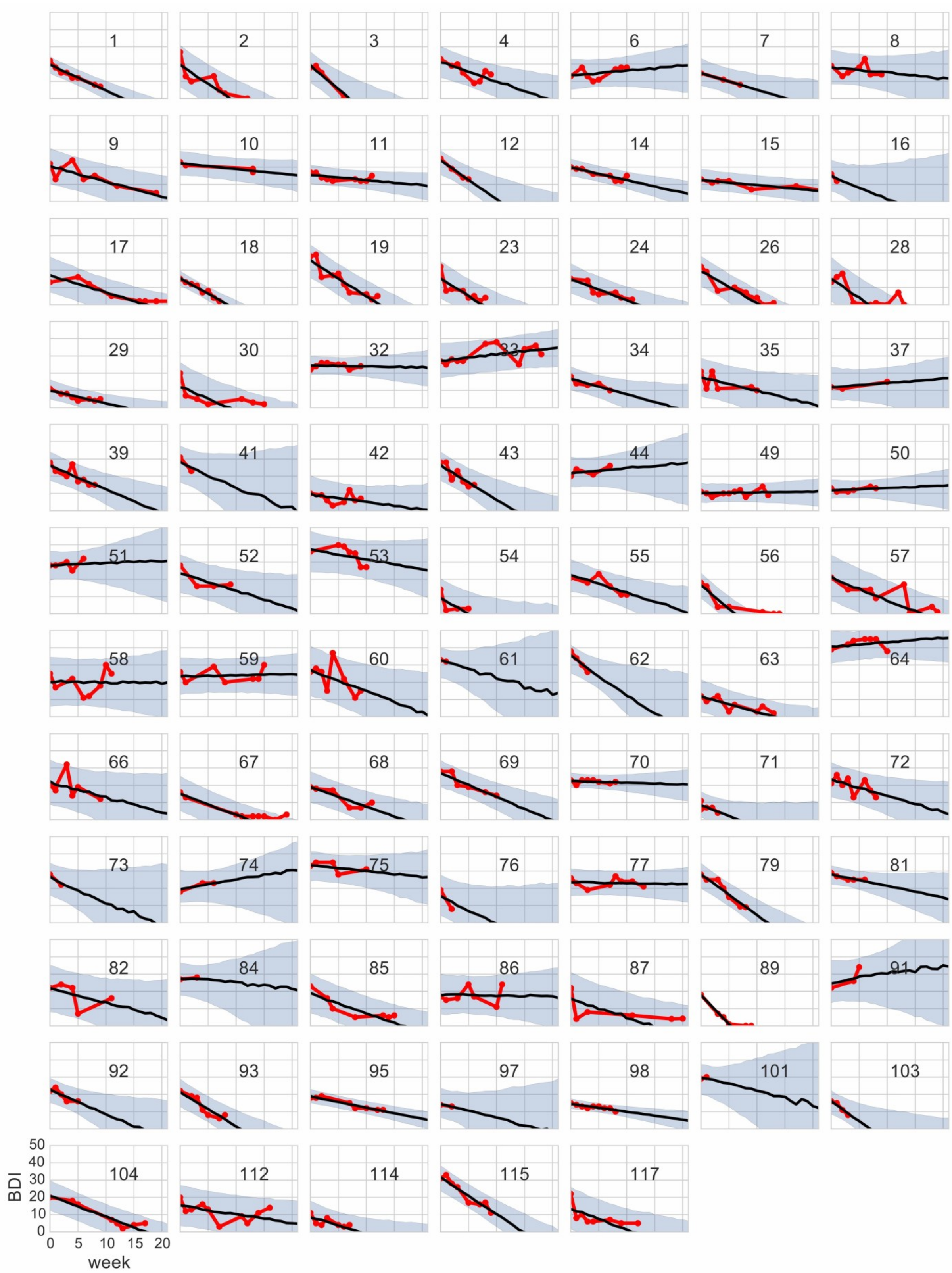

Figure S2. Posterior predictive plots for the linear model. Each plot is data from a single subject over time (in weeks). Red solid line is data, black line is the mode of the posterior distribution estimated from the Markov-chain Monte-Carlo samples, and the shaded area indicates the 95 % highest density interval.

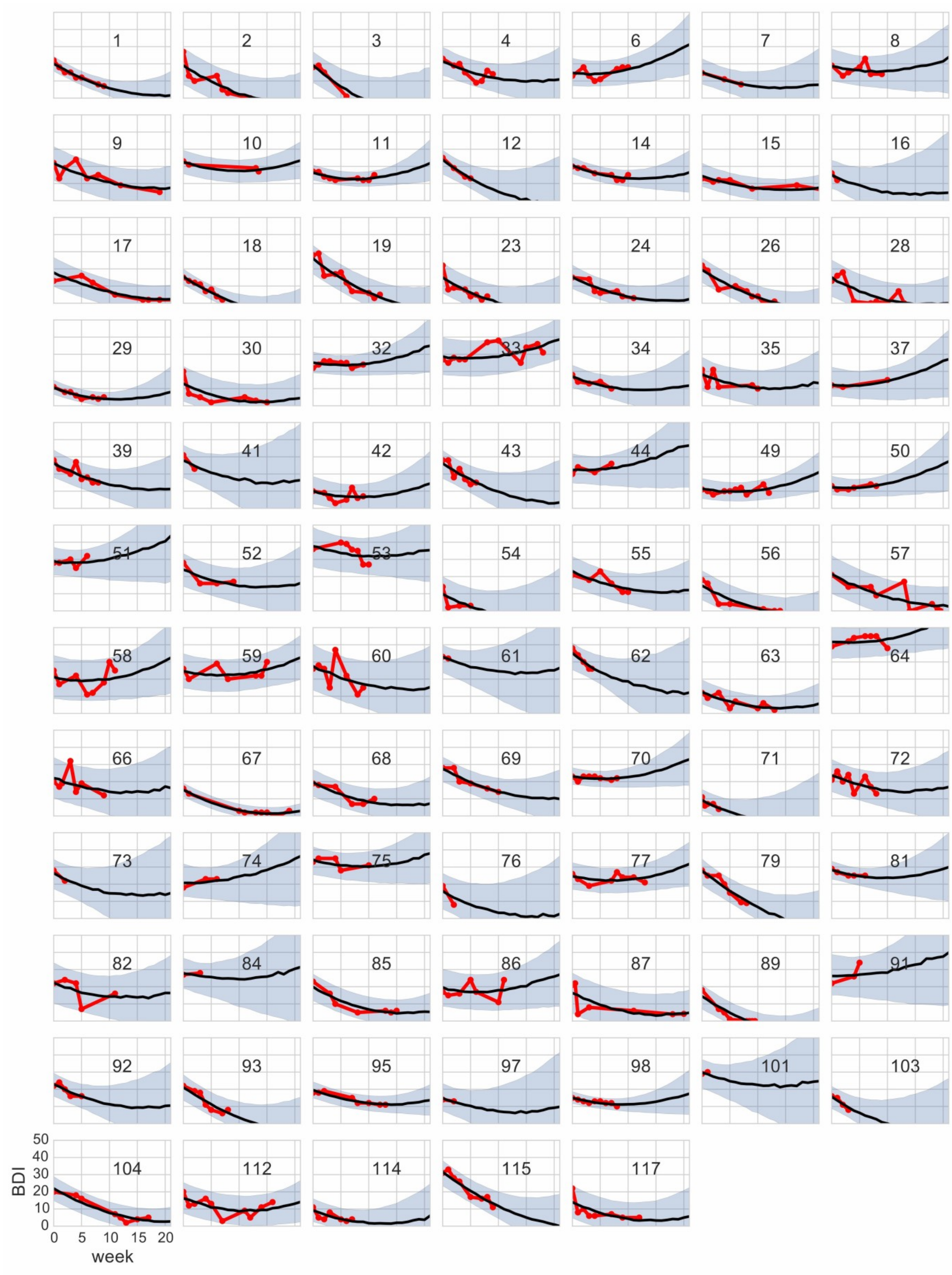

Figure S3. Posterior predictive plots for the quadratic model. Each plot is data from a single subject over time (in weeks). Red solid line is data, black line is the mode of the posterior distribution estimated from the Markov-chain Monte-Carlo samples, and the shaded area indicates the 95 % highest density interval.

Predicting probability of response. Response to depression treatment varies substantially across individuals [8]. Latent class approaches allow for the modeling of different growth trajectories across subgroups, and captures this unobserved heterogeneity in trajectories by employing a categorical latent variable with *k* classes [9, 10]. Class membership is initially unknown, but is inferred based on observed data, resulting in identified classes of individuals with more similar response patterns within each group than between groups [9]. Thus, different classes of individuals may vary around different mean growth curves with potentially unique forms and parameter values. This can be advantageous compared to conventional growth modeling which assumes that all individuals are drawn from the same population and estimates the average growth curve for this population [11]. Furthermore, covariates can be included in the model to predict class membership, and in this way individual characteristics predicting differential trajectories may be identified. Previous investigations have successfully employed latent class methods to identify different distributions for groups of responders and non-responders to treatment [12-14]. We therefore chose to fit a model which assumed two different distributions from which subject-level parameters could be drawn. Specifically, we modified the exponential (which yielded the best fit) model from above to read

$$b_i \sim N(\mu_{b,z}, \sigma_{b,z})$$

and let z be the result of a Bernoulli process with probability of response p_{resp}

$$z \sim \text{Bernoulli}\,(p_{resp})$$

which was informed by the individual values of the m predictor variables $x_{j,i}$ for subject i by means of a logistic regression

$$p_{resp} = \frac{1}{1+\exp(-(\beta_0 + \sum_{j=1}^{m} \beta_j x_{j,i}))}. \tag{4}$$

We standardized all variables x_j to z-values and used a unit-information prior on their

corresponding regression coefficients

$$\beta_i \sim N(0,1).$$

The group-level parameters received an unbiased, mildly informative prior as before

$$\mu_{b,z} \sim N(0,50)$$

and

$$\sigma_{b,z} \sim \text{Uniform}(0,50)$$

for all z . The label-switching problem was resolved by enforcing sequential ordering in each step of the MCMC sampling process [15].

To incorporate the idea that there is a class of non-responders and a class of responders, we also fitted a restricted version of this model where the mean of the non-responder group was fixed at zero, $\mu_{b,0} = 0$. The unrestricted two-class model did not improve the fit of the model (DIC = 2903), and the restricted version yielded a slightly better model fit (DIC = 2898). Qualitatively, the two models provide similar results. The estimate (mode of the posterior distribution) of the exponential slope of the non-responder group in the unrestricted model was 0.03 and its 95 % highest density interval (HDI) included zero [-0.005,0.07]. The mean exponential slope of the responder group was similar in the restricted (Mean = 0.11, HDI = [0.08,014]) and the unrestricted models (Mean = 0.12, HDI = [0.08,0.18]). We therefore use the restricted model as the better fitting model for subsequent analysis. The fit of this model is shown in Figure S1, and the distribution of the probability for being a responder (p_{resp}) across subjects is displayed in Figure S4. Note that the model appears to distinguish well between non-responders and responders. The impact of the predictor variables on group membership was analyzed using parameter estimates and odds-ratios.

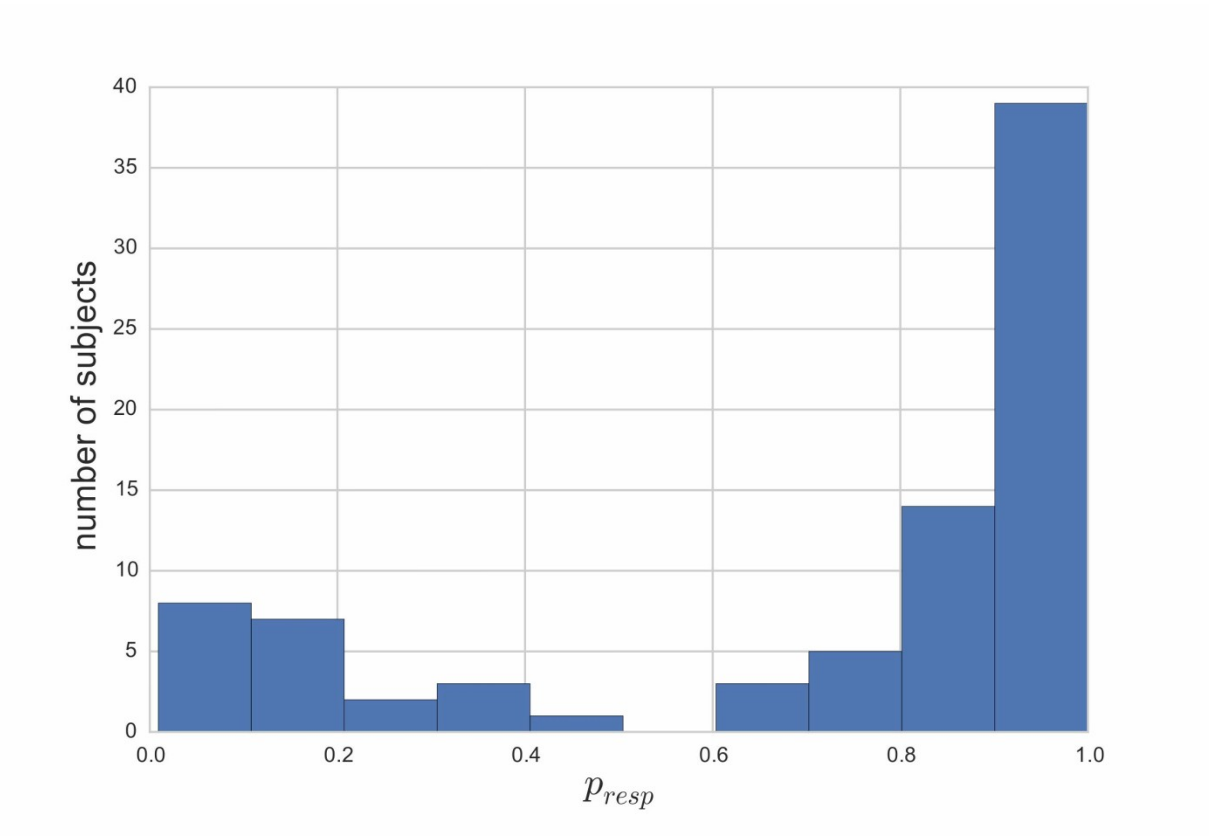

Figure S4. Distribution of p_{resp} across subjects. Note that the model appears to distinguish well between non-responders and responders.

Predicting the strength of response. In a next step, we aimed to explain variation in responsiveness. This analysis proceeded in two steps: First, we identified variables that correlate with the slope of the response. This is an alternative way to look at prediction of response, and it has the advantage of being more directly comparable to previous studies since latent-class approaches has not been widely used in the field. Second, we applied the same model specifically to the group of responsive patients (identified by the latent-class modeling) to find potentially modulating factors. This is a unique and more explorative analysis and expands on the other analyses by providing additional information about factors related to differential response among those who do show some level of improvement.

We modelled this situation by adding the subject-level covariates as linear predictors on the estimate of the first-level regression slope. Because changes of the slope parameter in

the exponential model are not reflected linearly (a unit change on a low slope parameter has strong impact while the same change on a higher slope parameter has less impact), we relied on the quadratic model for this approach. Even though the model had a slightly worse fit to the data, it produced qualitatively similar results as indicated by the posterior predictive plots (see Figure S3).

In this model, we let the linear slope coefficients b_i for subject i from Eq. (2) vary as a linear combination of the covariates

$$b_i = \alpha_0 + \sum_{j=1}^{m} \alpha_j x_{j,i} \tag{5}$$

where the coefficients α were estimated under the same prior distribution as in the responder-model. We fit this model twice, once to the whole group of patients, and once to the subgroup of responders (n = 61) as extracted by the latent-class model described in the previous section. Bayes factors for the α -coefficients were calculated using the Savage-Dickey ratio as detailed in Rouder and Morey[16].

Results

Descriptive statistics

Several variables were investigated as potential predictors of response. Figure S5 shows the distributions for the covariates, and Table S2 shows the means and standard deviations of BDI-II scores for baseline and follow-up (the last measurement occasion of each participant) stratified by each covariate.

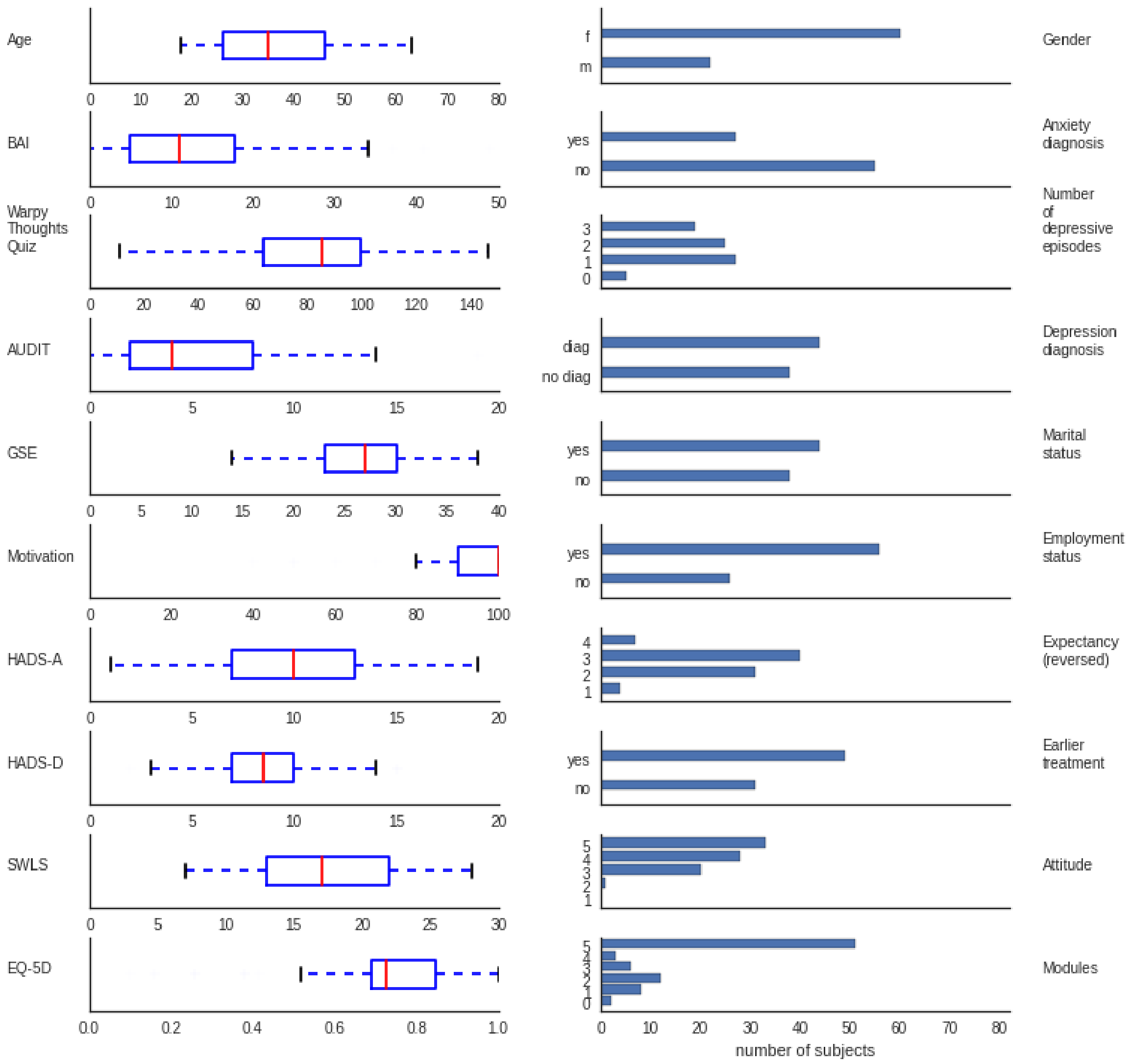

Figure S5. Summary of continuous (left) and categorical (right) predictor variables. Tukey box plots are shown for the continuous variables. The red lines indicate the median, and the boxes cover the area between the first and third quartile.

Table S2. Means and standard deviations of the BDI scores at baseline and follow-up stratified by each covariate (median-split for continuous variables). As there was no fixed posttreatment time-point, each individual's last BDI-II is used for follow-up.

Variable[a]	Categories	Baseline BDI-II			Last BDI-II		
		M	SD	n	M	SD	n
Gender	Male	18.8	5.6	22	8.8	6.4	22
	Female	22.2	6.7	60	14.9	10.3	60
Age	< Median	23.3	6.8	39	15.3	10.9	39
	≥ Median	19.5	5.9	43	11.4	8.3	43
Marital status	Married/ cohabiting	21.3	5.6	44	11.2	8.5	44
	Not married/ cohabiting	21.3	7.6	38	15.7	10.7	38
Work status	Paid job	21.0	5.5	56	11.9	8.9	56
	No paid job	21.9	8.6	26	16.3	11.0	26
Earlier treatment	No	20.7	6.3	31	12.7	10.5	31
	Yes	21.8	6.8	49	13.8	9.5	49
Depression diagnosis	No	18.6	5.9	38	11.0	8.3	38
	Yes	23.7	6.3	44	15.3	10.6	44
Number of depressive episodes	< Median	21.3	7.1	32	14.9	10.1	32
	≥ Median	21.7	6.6	44	12.7	9.8	44
Anxiety diagnosis	No	20.1	6.1	55	12.0	9.1	55
	Yes	23.8	6.9	27	15.8	10.8	27
Beck Anxiety Inventory	< Median	18.3	5.4	38	10.3	7.1	38
	≥ Median	23.9	6.5	44	15.9	11.0	44
HADS Depression	< Median	17.4	5.1	31	9.1	6.4	31
	≥ Median	24.1	6.1	48	15.8	10.9	48
HADS Anxiety	< Median	19.2	5.8	39	11.3	8.2	39
	≥ Median	23.8	6.6	40	15.1	11.2	40
Satisfaction with Life	< Median	24.4	6.6	33	18.5	10.8	33
	≥ Median	19.3	6.1	41	9.7	7.6	41
EQ-5D	< Median	22.8	6.8	36	15.7	9.9	36
	≥ Median	20.5	6.6	37	11.5	10.1	37

AUDIT	< Median	20.3	6.5	38	11.1	9.0	38
	≥ Median	22.2	6.7	43	15.4	10.2	43
Warpy Thoughts Quiz	< Median	19.1	5.1	40	9.3	7.9	40
	≥ Median	23.5	7.1	40	17.0	10.1	40
General Self Efficacy	< Median	23.5	6.4	40	16.1	10.2	40
	≥ Median	19.2	6.1	40	11.0	8.7	40
Motivation	< Median	22.5	5.5	25	12.5	8.1	25
	≥ Median	20.8	7.0	57	13.6	10.5	57
Expectancy	< Median	20.7	6.3	35	12.4	9.2	35
	≥ Median	21.8	6.8	47	13.9	10.2	47
Attitude	< Median	21.8	6.4	21	13.4	8.4	21
	≥ Median	21.1	6.7	61	13.2	10.3	61
Number of modules	< Median	22.0	7.0	32	16.8	9.4	32
	≥ Median	20.9	6.3	50	11.0	9.4	50

[a]AUDIT: Alcohol Use Disorders Identification Test; EQ-5D: EuroQol 5-Dimension Self-Report Questionnaire; HADS-A: Hospital Anxiety and Depression Scale-anxiety subscale; HADS-D:Hospital Anxiety and Depression Scale-depression subscale.

Variation in response within the responder group

Variation in responsiveness was analyzed separately for the full sample of participants and for the subgroup of responders (n = 61) extracted by the latent-class model. Once the analysis was restricted to the group of responders, other variables predicting how strongly a participant will respond to treatment emerged (see Table S3). Being female and scoring higher on the anxiety subscale of HADS were factors with high odds for having a positive impact on treatment effect. Previous treatment, reporting a positive attitude towards Internet-based treatment, having a paid job and older age had the highest odds for predicting poorer response within the responder group.

Table S3: Posterior mode, highest density interval (HDI) and odds-ratios for the α-coefficients predicting the strength of the response. The α –coefficients are the group-level regression coefficients on the slope of the treatment effect in the quadratic model (see Equation 5). The odds-ratios indicate the probability that each covariate has a positive/negative impact relative to the probability of the opposite (+ positive effect, − negative effect), but do not indicate the strength of this effect.

Variable[a]	Posterior Mode(HDI)	OR $\|\alpha_i\| > 0$
Warpy Thoughts Quiz	0.09 (-0.13, 0.35)	4.24^{+}
Motivation	0.15 (-0.17, 0.43)	4.21^{+}
GSE	0.09 (-0.25, 0.41)	2.12^{+}
EQ-5D	0.07 (-0.17, 0.30)	2.44^{+}
Earlier treatment	−0.25 (-0.46, -0.01)	52.82^{-}
AUDIT	0.09 (-0.09, 0.28)	5.44^{+}
HADS-D	0.11 (-0.18, 0.40)	3.68^{+}
Age	−0.17 (-0.39, 0.05)	14.76^{-}
Attitude	−0.22 (-0.44, 0.03)	24.44^{-}
BAI	−0.06 (-0.40, 0.23)	2.26^{-}
Gender	0.13 (-0.07, 0.31)	8.84^{+}
HADS-A	0.31 (0.01, 0.61)	39.29^{+}
Depression diagnosis	0.04 (-0.21, 0.29)	1.51^{+}
Anxiety diagnosis	0.12 (-0.13, 0.39)	5.32^{+}
Expectancy (reversed)	0.05 (-0.20, 0.30)	1.95^{+}
Employment status	−0.16 (-0.38, 0.06)	13.15^{-}
Marital status	−0.03 (-0.25, 0.20)	1.39^{-}
Modules	0.01 (-0.24, 0.27)	1.27^{+}
Number of depressive episodes	−0.00 (-0.23, 0.20)	1.14^{-}
SWLS	−0.06 (-0.36, 0.22)	2.30^{-}

[a]AUDIT: Alcohol Use Disorders Identification Test; BAI: Beck Anxiety Inventory; EQ-5D: EuroQol 5-Dimension Self-Report Questionnaire; GSE: General Self-efficacy Scale; HADS-A: Hospital Anxiety and Depression Scale-anxiety subscale; HADS-D:Hospital Anxiety and Depression Scale-depression subscale; SWLS: Satisfaction With Life Scale.

Bayes factors quantify the strength of evidence for the null-hypothesis (the covariate does not affect treatment response) and for the alternative hypothesis (the covariate affects

response to treatment). The results are presented in Table S4 for the group of responders.

Table S4: Bayes factors quantifying the evidence for H_1 over H_0 (BF_{10}) for the group of responders. Variables are sorted with respect to its Bayes factor in ascending order. The null-hypothesis is that the predictor does not have an impact on treatment efficacy (H_0: $\alpha_i = 0$) and the alternative is that it does have an effect (H_1: $\alpha_i \neq 0$). BF_{10} is the odds for H_1 divided by the odds for H_0.

Variable[a]	BF_{10}	Evidence for
Number of depressive episodes	0.19	H_0 : substantial
Employment status	0.20	H_0: substantial
Motivation	0.23	H_0 : substantial
Depression diagnosis	0.24	H_0 : substantial
EQ-5D	0.24	H_0 : substantial
Attitude	0.28	H_0 : substantial
AUDIT	0.28	H_0 : substantial
SWLS	0.30	H_0 : substantial
Marital status	0.31	H_0 : substantial
Gender	0.32	H_0 : substantial
Expectancy	0.48	H_0 : anecdotal
Earlier treatment	0.54	H_0 : anecdotal
Modules	0.88	H_0 : anecdotal
Age	1.16	H_1 : anecdotal
HADS-D	1.49	H_1 : anecdotal
BAI	2.42	H_1 : anecdotal
GSE	2.76	H_1 : anecdotal
Warpy Thoughts Quiz	3.71	H_1 : substantial
Anxiety diagnosis	4.43	H_1 : substantial
HADS-A	21.51	H_1 : strong

[a]AUDIT: Alcohol Use Disorders Identification Test; BAI: Beck Anxiety Inventory; EQ-5D: EuroQol 5-Dimension Self-Report Questionnaire; GSE: General Self-efficacy Scale; HADS-A: Hospital Anxiety and Depression Scale-anxiety subscale; HADS-D=Hospital Anxiety and Depression Scale-depression subscale; SWLS: Satisfaction With Life Scale.

The analysis indicated that there was substantial evidence that most covariates did not influence the treatment effect (see Table S4). There was, however, substantial evidence for a

positive effect of Warpy thoughts and having an anxiety diagnosis, and strong evidence for a positive effect of higher scores on the anxiety subscale of the HADS.

Discussion

In a supplemental analysis the variation in responsiveness was explored within the sample of responders by analyzing whether predictors affected the slope of response. Results indicated that higher scores on the Warpy Thoughts Quiz and higher levels of baseline anxiety measured with HADS, as well as the presence of a comorbid anxiety disorder, predicted better treatment effects among responders.

Investigating predictors of the strength of response among participants categorized as being responders (showing some improvement during treatment) within the latent-class analysis may yield different results compared to analyses focusing on the whole sample of participants.

Interestingly, compared to the analysis including the whole sample, within the responder group the opposite pattern was found for Warpy Thoughts with the Bayes factor indicating substantial evidence for a positive effect of this variable. This suggests that among participants who do respond to treatment, perhaps due to other facilitative characteristics (such as having a partner), those with higher scores on the Warpy Thought Quiz display more improvement. This is difficult to explain, but given that cognitive restructuring is a central topic in the MoodGYM program one can speculate that those with more dysfunctional thinking, albeit not too much, are more strongly motivated to regulate their cognitive processes to achieve a much wanted emotional relief. Further studies are required to explore such a hypothesis.

The strongest predictor of superior response within the responder group was anxiety. The Bayes factors indicated strong and substantial evidence for an effect of anxiety measured with HADS and the presence of an anxiety diagnosis, respectively. This suggests that among participants showing some level of response, those having an anxiety diagnosis or reporting higher levels of anxiety on the HADS, experienced more improvement. As these results pertain only to the responder group they are not directly comparable to previous results from analyses focusing on full samples. Some recent studies of CBT delivered face-to-face found that patients with comorbid anxiety showed more rapid change early in treatment [17-19], as well as greater overall change [20]. Proposed mechanisms for this finding was that individuals with anxiety show a larger response to common factors such as the warmth and empathy of a therapist [17], or that anxious activation may help mobilize the individual towards change [20]. In the present study a similar pattern was evident when analyzing the responder-group separately, but not in the analysis of the full sample as would be expected based on these past studies. It may also be noted that this effect was evident for anxiety measured with HADS, on which the intervention had significant effects, and not for BAI for which the intervention did not show effects [21]. Why this effect was only found among responders and whether it is related to an enhanced effect of common factors for this group of patients or to other mechanisms, remains to be elucidated by future studies.

The present analysis was of an exploratory nature, and results can mainly be viewed as hypothesis to be investigated in further studies. However, the analysis indicates that the variables that predict responsiveness within a class of responders may differ from the variables predicting class membership (predicting response vs nonresponse) and the variables that predict responsiveness in a sample consisting of both responders and non-responders. The study of subgroups of responders and their characteristics is an interesting approach to gain knowledge about differential treatment response.

References

1. Gelman A, Carlin J, Stern H, Dunson D, Vehtari A, Rubin DB. Bayesian data analysis. 2rd edition. Boca Raton,FL: CRC Press, Taylor & Francis Group; 2013. ISBN: 9781439840955.
2. Gelman A, Shalizi CR. Philosophy and the practice of Bayesian statistics. Brit J Math Stat Psy 2013;66(1):8-38. PMID:22364575.
3. Kruschke J. Doing Bayesian data analysis: a tutorial introduction with R. Oxford: Academic Press; 2010. ISBN: 9780123814852.
4. Plummer M. JAGS: a program for analysis of Bayesian graphical models using Gibbs sampling. Proceedings of the 3rd International Workshop on Distributed Statistical Computing 2003 20-22. Vienna, Austria.
5. Gelman A, Rubin DB. Inference from iterative simulation using multiple sequences. Stat Sci 1992;7(4):457-472. doi:10.2307/2246093.
6. Spiegelhalter DJ, Best NG, Carlin BP, Van Der Linde A. Bayesian measures of model complexity and fit. J R Stat Soc Series B Stat Methodol 2002;64(4):583-639. doi:10.1111/1467-9868.00353.
7. Pratte MS, Rouder JN. Assessing the dissociability of recollection and familiarity in recognition memory. J Exp Psychol Learn Mem Cogn 2012;38(6):1591-1607. PMID:22563630.
8. Simon GE, Perlis RH. Personalized medicine for depression: can we match patients with treatments? Am J Psychiatry 2010;167(12):1445-1455. PMID:20843873.
9. Jung T, Wickrama KAS. An introduction to latent class growth analysis and growth mixture modeling. Soc Personal Psychol Compass 2008;2(1):302-317. doi:10.1111/j.1751-9004.2007.00054.x.
10. Tofighi D, Enders CK. Identifying the correct number of classes in growth mixture models. In: Hancock GR and Samuelsen KM, editor. Advances in latent variable mixture models. Greenwhich, CT: Information Age; 2007. p. 317-341. ISBN: 9781593118488.
11. Muthén B. Latent variable analysis: growth mixture modeling and related techniques for longitudinal data. In: Kaplan D, editor. The SAGE handbook of quantitative methodology for the social sciences. Newbury Park, CA: SAGE Publications; 2004. p. 345-368. ISBN: 9780761923596
12. Lutz W, Stulz N, Köck K. Patterns of early change and their relationship to outcome and follow-up among patients with major depressive disorders. J Affect Disord 2009;118(1-3):60-68. PMID:19217669.
13. Stulz N, Lutz W, Leach C, Lucock M, Barkham M. Shapes of early change in psychotherapy under routine outpatient conditions. J Consult Clin Psychol 2007;75(6):864-874. PMID:18085904.
14. Muthén B, Brown HC, Hunter AM, Cook IA, Leuchter AF. General approaches to analysis of course: applying growth mixture modeling to randomized trials of depression medication. In: Shrout PE, Keyes K , and Ornstein K, editor. Causality and psychopathology: finding the determinants of disorders and their cures. New York, NY: Oxford University Press; 2010. p. 159-177. ISBN: 9780199754649.
15. Stephens M. Dealing with label switching in mixture models. J R Stat Soc Series B Stat Methodol 2000;62(4):795-809. doi:10.1111/1467-9868.00265.
16. Rouder JN, Morey RD. Default Bayes Factors for model selection in regression. Multivar Behav Res 2012;47(6):877-903. doi:10.1080/00273171.2012.734737.

17. Forand NR, Derubeis RJ. Pretreatment anxiety predicts patterns of change in cognitive behavioral therapy and medications for depression. J Consult Clin Psychol 2013;81(5):774-782. PMID:23647285.
18. Smits JAJ, Minhajuddin A, Thase ME, Jarrett RB. Outcomes of acute phase cognitive therapy in outpatients with anxious versus nonanxious depression. Psychother Psychosom 2012;81(3):153-160. PMID:22398963.
19. Kashdan TB, Roberts JE. Comorbid social anxiety disorder in clients with depressive disorders: predicting changes in depressive symptoms, therapeutic relationships, and focus of attention in group treatment. Behav Res Ther 2011;49(12):875-884. PMID:22018535.
20. Forand NR, Gunthert KC, Cohen LH, Butler AC, Beck JS. Preliminary evidence that anxiety is associated with accelerated response in cognitive therapy for depression. Cogn Ther Res 2011;35(2):151-160. doi:10.1007/s10608-010-9348-5.
21. Høifødt RS, Lillevoll KR, Griffiths KM, Wilsgaard T, Eisemann M, Waterloo K, et al. The clinical effectiveness of Web-based cognitive behavioral therapy with face-to-face therapist support for depressed primary care patients: randomized controlled trial. J Med Internet Res 2013;15(8):e153. PMID:23916965.
