# Supplementary material for: Predictors of Response to Web-Based Cognitive Behavioral Therapy With High-Intensity Face-to-Face Therapist Guidance for Depression: A Bayesian Analysis
Source: J Med Internet Res. 2015 Sep 2;17(9):e197. doi: 10.2196/jmir.4351 (PMC4642793; doi:10.2196/jmir.4351)
Supplement: Multimedia Appendix 2 [file jmir_v17i9e197_app2.pdf]

CONSORT-EHEALTH Checklist V1.6.2 Report

(based on CONSORT-EHEALTH V1.6), available at [http://tinyurl.com/consort-ehealth-v1-6].

4351

Date completed

7/3/2013 11:25:58

by

Ragnhild Sørensen Høifødt

The clinical effectiveness of web-based cognitive behavioural therapy with face-to-face therapist support for depressed primary care patients: A randomised controlled trial

TITLE

1a-i) Identify the mode of delivery in the title

“The clinical effectiveness of web-based cognitive behavioural therapy with face-to-face therapist support for depressed primary care patients”

1a-ii) Non-web-based components or important co-interventions in title

“The clinical effectiveness of web-based cognitive behavioural therapy with face-to-face therapist support for depressed primary care patients”

1a-iii) Primary condition or target group in the title

“The clinical effectiveness of web-based cognitive behavioural therapy with face-to-face therapist support for depressed primary care patients”

ABSTRACT

1b-i) Key features/functionalities/components of the intervention and comparator in the METHODS section of the ABSTRACT

“Participants were allocated to a treatment condition comprising 6 weeks of therapist assisted web-based CBT, or to a 6 weeks delayed treatment condition. The intervention included the Norwegian version of the MoodGYM program, brief face-to-face support from a psychologist, and reminder e-mails.”

1b-ii) Level of human involvement in the METHODS section of the ABSTRACT

“The intervention included the Norwegian version of the MoodGYM program, brief face-to-face support from a psychologist, and reminder e-mails.”

1b-iii) Open vs. closed, web-based (self-assessment) vs. face-to-face assessments in the METHODS section of the ABSTRACT

“Participants were recruited from primary care recruited from primary care” “The intervention included the Norwegian version of the MoodGYM program, brief face-to-face support from a psychologist, and reminder e-mails.”

“As the primary outcome measure depression symptoms were measured by means of the Beck Depression Inventory-II (BDI-II). Secondary outcome measures included the Beck Anxiety Inventory (BAI), the Hospital Anxiety and Depression Scale (HADS), the Satisfaction with Life Scale (SWLS) and the EuroQol Group 5-Dimension Self-Report Questionnaire (EQ-5D). All outcomes were based on self-report and were assessed at baseline, post-intervention and at 6 months follow-up.”

Elaboration from the main body of the manuscript: “The therapists were not blind to the participants` condition. However, steps were taken to blind the evaluation of outcomes by ensuring that post-tests were performed by a research assistant unaware of the participants’ allocation assignment.”

1b-iv) RESULTS section in abstract must contain use data

“Post-intervention measures were completed by 37 (71 %) and 47 (87 %) of the 52 participants in the intervention and 54 participants in the delayed treatment group, respectively. Linear mixed-models analyses revealed significant difference in time trends between the groups for the BDI-II, (P = .002), for HADS depression and anxiety subscales (P < .001 and P = .001, respectively), and for the SWLS (P < .001). No differential group effects were found for the BAI and the EQ-5D. In comparison to the control group significantly more participants in the intervention group experienced recovery from depression as measured by the BDI-II. Sixty per cent (31 of 52 participants) adhered to the treatment program, and overall treatment satisfaction was high. The reduction of depression and anxiety symptoms was largely maintained at 6-months follow-up, and positive gains in life satisfaction were partly maintained.”

1b-v) CONCLUSIONS/DISCUSSION in abstract for negative trials

“The intervention combining MoodGYM and brief therapist support can be an effective treatment for depression in a sample of primary care patients. The intervention alleviates depressive symptoms and has significant positive effects on anxiety symptoms and satisfaction with life. Moderate rates of non-adherence and predominately positive evaluations of the treatment, also indicates the acceptability of the intervention. The intervention could have a potential for use in a stepped-care approach, but remains to be tested in regular primary health care.”

INTRODUCTION

2a-i) Problem and the type of system/solution

“The majority of patients with psychological problems will receive most or all of their mental health care in primary care, and findings suggest that many patients prefer to consult their GP for treatment of depression [2, 37-39]. Clinical practice guidelines primarily recommend treating mild to moderate depression using psychosocial interventions [40, 41], and this is also in accordance with reported patient preferences [42-44]. Despite this, structured psychological interventions are infrequently delivered in general practice [45-47], due to time constraints in general practice [48-50] and a lack of knowledge and competence among GPs in the delivery of evidence-based psychological interventions [50, 51]. The use of CBT-based self-help resources could be a way to improve the delivery of psychological interventions in general practice.”

2a-ii) Scientific background, rationale: What is known about the (type of) system

“A substantive body of research shows that internet-based CBT can be an efficacious treatment for depression [e.g.11, 12-14].” “An increasing amount of research has pointed to the importance of support in internet-based interventions, with interventions offering some degree of support from a professional during treatment generally showing substantially larger treatment effects than interventions involving little or no professional support [25-27].” “Studies have demonstrated the effectiveness of MoodGYM in reducing symptoms of depression and anxiety [19, 23, 24, 28, 32-34]..” “However, few previous trials have investigated the effect of MoodGYM combined with therapist support.” “The use of CBT-based self-help resources could be a way to improve the delivery of psychological interventions in general practice. “ “The current study was designed to trial a procedure for depression treatment which could be suitable for implementation in general practice. The project was planned as the first phase of research for this treatment, with the second phase focusing on further evaluation carried out in everyday general practice.” For this first evaluation of the present intervention a delayed treatment control condition was considered an acceptable comparator. The results of this comparison will give preliminary evidence of the effect of the intervention for this patient group. Further studies will be needed to evaluate the effect compared to other treatments.

METHODS

3a) CONSORT: Description of trial design (such as parallel, factorial) including allocation ratio

“The aim was to evaluate the effectiveness and acceptability of a guided self-help intervention combining the MoodGYM program with brief face-to-face therapist support in a sample of primary care patients with mild to moderate symptoms of depression. The primary hypothesis was that therapist supported web-based CBT would lead to a larger reduction in depressive symptoms than the control condition.”

3b) CONSORT: Important changes to methods after trial commencement (such as eligibility criteria), with reasons

“During the first months of the study the protocol was changed by extending the inclusion criterion on BDI-II (from including participants with scores between 14 and 29) to include participants with scores between 10 and 40. This change was due to insufficient recruitment and to the clinical appraisal that patients with scores above 30 could possibly benefit from the treatment, based on their daily functioning and motivation. In addition, their depression was too mild to assure them other public treatment options. Furthermore, several patients with a BDI-II score below 14 reported a need for treatment.”

3b-i) Bug fixes, Downtimes, Content Changes

There were no such changes influencing the intervention or the design during the trial.

4a) CONSORT: Eligibility criteria for participants
“Study inclusion criteria were: (1) 18 – 65 years of age, (2) access to the Internet, and (3) a score between 14 and 29 on the Beck Depression Inventory-II (BDI-II), indicating mild to moderate symptoms of depression.” “Individuals currently undergoing CBT were excluded, while individuals who used antidepressant medication were stabilised for one month prior to evaluation of diagnostic eligibility.” “Medical or psychiatric comorbidities only restricted inclusion when there was a need for immediate treatment of these comorbid conditions (suicidal ideation, current psychosis) or where the conditions were expected to markedly interfere with treatment of the depressive condition (alcohol or drug use disorders).”

4a-i) Computer / Internet literacy
Computer/ Internet literacy was not an eligibility criterion and is therefore not addressed in the methods section. Internet access is explicitly stated as an eligibility criterion.

4a-ii) Open vs. closed, web-based vs. face-to-face assessments:
“Participants were recruited from GPs, primary care nurses and from waitlists of primary care referrals at two psychiatric outpatient clinics.” “Participants were screened for inclusion through a face-to-face session.” “After each module participants received brief face-to-face support (15 – 30 minutes).”

4a-iii) Information giving during recruitment
“Local GPs and primary care nurses were informed about the study both verbally at practice meetings and through written information. They provided patients who they considered to be mildly to moderately depressed based on clinical appraisal and/or screening instruments with written information about the project. Potentially eligible patients on waitlists for psychiatric outpatient treatment were identified by clinic staff and subsequently received information by postal mail from the research group.“ Trial information is included as appendix.

4b) CONSORT: Settings and locations where the data were collected
“The study was conducted at the Department of Psychology at the University of Tromsø where a small self-help outpatient clinic was established.”

4b-i) Report if outcomes were (self-)assessed through online questionnaires
“Assessments of symptoms of depression, anxiety and quality of life using paper-and-pencil questionnaires, were completed by all participants at baseline, post-treatment and at 6-months post-treatment (online questionnaires).”

4b-ii) Report how institutional affiliations are displayed
“The written information used a logo to mark the affiliation to the University of Tromsø (local academic institution)”

5) CONSORT: Describe the interventions for each group with sufficient details to allow replication, including how and when they were actually administered

5-i) Mention names, credential, affiliations of the developers, sponsors, and owners
“The MoodGYM was originally developed at the Australian National and they provided access to the MoodGYM in the trial”
“KG is one of the authors of the MoodGYM intervention evaluated in the trial and work for The Australian National University who provide free access to the MoodGYM websites.”” ME and KW contributed in the process of translating MoodGYM into Norwegian.”

5-ii) Describe the history/development process
The web-based program (MoodGYM) is well established, and both the English and Norwegian versions are tested in previous trials. These and work describing the intervention in detailed is cited in the introduction.

5-iii) Revisions and updating
“The guided self-help intervention involved the Norwegian version of the web-based CBT program MoodGYM (version 3)”. The intervention is well established, and no major changes were made during the evaluation process, only minor text editing.

5-iv) Quality assurance methods
The MoodGYM program is well established, and work describing the intervention in detail is cited in the introduction and methods sections.

5-v) Ensure replicability by publishing the source code, and/or providing screenshots/screen-capture video, and/or providing flowcharts of the algorithms used
The MoodGYM program is well established, and work describing the intervention in detail is cited in the introduction and methods sections.
The URL is also cited in the manuscript. Readers can access the program free of charge at www.moodgym.anu.edu.au.

5-vi) Digital preservation
“The guided self-help intervention involved the Norwegian version of the web-based CBT program MoodGYM(version 3; www.moodgym.anu.edu.au)”. “The program consists of 5 modules and a personal workbook containing exercises and assessments.” The URL with the archived login page is provided in the manuscript, as well as screen shots from the program. Readers can access the program free of charge.

5-vii) Access
“MoodGYM is a free web-based CBT program.” “In the first session after screening, participants were introduced to the program, received their trial username and password, and were instructed to work at home with one module each week. “ Readers can access the program free of charge.

5-viii) Mode of delivery, features/functionalities/components of the intervention and comparator, and the theoretical framework
“The guided self-help intervention involved the Norwegian version of the web-based CBT program MoodGYM(version 3; www.moodgym.anu.edu.au ”. “The program consists of 5 modules and a personal workbook containing exercises and assessments. Module 1 through 3 focus on the cognitive model, typical patterns of dysfunctional thinking and exercises to identify and restructure dysfunctional thinking, as well as behavioural strategies to increase engagement in positive activities. Module 4 focuses on stress and stress reduction and introduces relaxation techniques. Module 5 covers simple problem solving and typical responses to broken relationships. Each module takes approximately 45 to 60 minutes to work through.” “Participants in both groups were free to access usual primary care treatment, which could include antidepressant medication, informal supportive therapy or referral to ordinary specialist mental health services.“

5-ix) Describe use parameters
“Participants were instructed to work at home with one module each week.” “The therapists focused on motivating the participants to adhere to the treatment plan and aimed to meet participants weekly and to complete the intervention over 7 weeks. However, the interval between sessions and the number of sessions were allowed to vary somewhat to provide flexibility in meeting individual needs.” The participants could work at their own pace, ad libitum.

5-x) Clarify the level of human involvement
“After each module participants received face-to-face support (15 – 30 minutes) from a psychologist. The number of sessions was allowed to vary somewhat to provide flexibility in meeting individual needs.”

5-xi) Report any prompts/reminders used
“Between sessions participants received tailored e-mails aiming to motivate them to work with the self-help program. The e-mails introduced the next module, and some contained brief advice on how to overcome depressive symptoms, e.g. the importance of behavioural activation.”

5-xii) Describe any co-interventions (incl. training/support)
“After each module participants received face-to-face support (15 – 30 minutes). The therapists followed a guideline script with three compulsory topics for every consultation: (1) Monitoring of depression symptoms and discussion of changes, (2) Focus on the important topics and exercises covered by each module and the participants’ experiences of working with this, (3) Introduce the next module and motivate participants to adhere to the treatment plan. . The main focus of the therapist was on reinforcing the efforts made by participants and helping them to relate to the material and to incorporate the use of techniques from the program into their everyday living. If time permitted, participants could also bring up other topics they considered important in relation to their depression. In the concluding session the experiences and outcomes of treatment were discussed.”

6a) CONSORT: Completely defined pre-specified primary and secondary outcome measures, including how and when they were assessed

“The primary outcome measure was the Beck Depression Inventory-II. The secondary outcome measures comprised the Beck Anxiety Inventory, the Hospital Anxiety and Depression Scale and two measures of quality of life (Satisfaction With Life Scale, EQ-5D), as well as a measure of treatment satisfaction. Assessments of symptoms of depression, anxiety and quality of life using paper-and-pencil questionnaires, were completed by all participants at baseline, post-treatment and at 6-months post-treatment (online questionnaires). The control group also completed these inventories before entering online treatment (post-waiting). BDI-II was administered before every consultation during the intervention phase.”

6a-i) Online questionnaires: describe if they were validated for online use and apply CHERRIES items to describe how the questionnaires were designed/deployed

Computerised or online versions of all measures have been used in previous studies, in which high correlations and comparable psychometric properties between the two formats have been found [58-61, 66, 69, 70, 74, 75, 80]. The online questionnaires were distributed by e-mail, and respondents were directed to a web-page where they filled-out the questionnaire. All online interactions used Secure Sockets Layer- encryption (SSL-encryption), and the Norwegian Data Inspectorate`s security regulations regarding data storage were adhered to. A hidden identity-functionality was used so that responses could not be linked to names or e-mail addresses, and thus all questionnaires were identifiable by unique ID-numbers only. Duplicate entries were prevented by not allowing respondents to display the survey a second time once they had filled it in. The design of the online questionnaires was identical to the original design of the paper-and-pencil versions with regard to instructions, number of items per page and number of pages. Items were not randomised or alternated. Adaptive questioning was used for questions regarding health service use, work status and sick leave. All items, except for adaptive items, were mandatory, and respondents were not allowed to return to review or change previous questions. The functionality and usability of the questionnaires was tested by the research group before fielding the questionnaires, but not formally tested on a wider range of users. “The use of different administration formats for the assessments of the treatment phase and follow-up, paper-and-pencil versus online questionnaires, respectively, can potentially introduce measurement bias. Although the two formats correlate highly, one study indicated a significant difference in mean scores on the BDI-II and BAI, which makes switching of formats problematic [58]. Despite this limitation, the results should not be considered weakened for most measures, since the direction of differences has generally suggested that online versions tend to inflate estimations of symptom severity and lower ratings of quality of life [58, 70, 75], with the exception of BAI, where Carlbring et al [58] found that means on the online version were lower compared to the paper-and-pencil version. The reliability of the 6-months follow-up results for the BAI may therefore be limited.”

6a-ii) Describe whether and how “use” (including intensity of use/dosage) was defined/measured/monitored

“Treatment variables included module completion, number of sessions, treatment duration, session duration (in minutes; not including screening) and total time spent by therapists (time spent besides the consultations were not registered). User data on module completion was registered online and was denoted by a number between 0 and 4, with 0 indicating no use and 4 indicating completion of the module.”

6a-iii) Describe whether, how, and when qualitative feedback from participants was obtained

Qualitative feedback from participants in the intervention group was collected through qualitative interviews after treatment. The results are presented in separate papers.

6b) CONSORT: Any changes to trial outcomes after the trial commenced, with reasons

“The quality of life measures were included during the initial phase of the trial due to the consideration that the extension of outcomes beyond symptom measures would strengthen the study.”

7a) CONSORT: How sample size was determined

7a-i) Describe whether and how expected attrition was taken into account when calculating the sample size

“A median drop-out rate of between 17 and 19 % has been reported for computerised or web-based treatment programs [53, 54]. With 20 % expected drop-out, a total sample size of 108 was required to gain sufficient power, yielding group-sizes of 54 participants.”

7b) CONSORT: When applicable, explanation of any interim analyses and stopping guidelines

No interim analyses. No explicit stopping guidelines. Symptom levels were discussed at every support session with the psychologist. In the case of significant worsening of symptoms necessitating immediate attention (f.ex. suicidality), the participant´s GP would be contacted (with consent from the participant) and the patient would be referred to specialised mental health services for more extensive treatment.

8a) CONSORT: Method used to generate the random allocation sequence

“A computerised random number generator randomised ID-numbers to the two groups (generated by KL). Eligible participants were given ID-numbers following a chronological sequence.” ID-numbers were divided between the two therapists in blocks ensuring approximately equal numbers of participants from each group.

8b) CONSORT: Type of randomisation; details of any restriction (such as blocking and block size)

“To ensure equal group sizes blocked randomisation with variable blocks sizes were used.”

9) CONSORT: Mechanism used to implement the random allocation sequence (such as sequentially numbered containers), describing any steps taken to conceal the sequence until interventions were assigned

“The present study has several limitations that need to be addressed. First, the lack of allocation concealment introduced a risk of bias that may have inflated the treatment effects.”

10) CONSORT: Who generated the random allocation sequence, who enrolled participants, and who assigned participants to interventions

“A computerised random number generator randomised ID-numbers to the two groups (generated by KL). Screening, enrolment and treatment were carried out by two clinical psychologists (RSH and KL).”

11a) CONSORT: Blinding - If done, who was blinded after assignment to interventions (for example, participants, care providers, those assessing outcomes) and how

11a-i) Specify who was blinded, and who wasn’t

“Patients could not be blinded to group assignment, but were blinded to the status of the waitlist as a control condition. The therapists were not blind to the participants` condition. However, steps were taken to blind the evaluation of outcomes by ensuring that post-tests were performed by a research assistant unaware of the participants’ allocation assignment.”

11a-ii) Discuss e.g., whether participants knew which intervention was the “intervention of interest” and which one was the “comparator”

As we used a delayed treatment control group participants could not be blind to whether or not they received the intervention. “Patients could not be blinded to group assignment, but were blinded to the status of the waitlist as a control condition.”

11b) CONSORT: If relevant, description of the similarity of interventions

Not relevant. Waitlist control was used in the trial.

12a) CONSORT: Statistical methods used to compare groups for primary and secondary outcomes

“Differences between the conditions on baseline characteristics were examined by performing chi-square tests for categorical variables and one-way analysis of variance (ANOVA) for continuous variables.” “Effects were tested by performing linear mixed-models analysis using the restricted maximum likelihood estimation (REML) procedure and an unstructured covariance matrix. Since linear mixed-models analysis can handle incomplete data, no procedure for imputation of missing data was utilised in the analysis [93]. For the analysis of BDI-II during the treatment phase random intercepts across participants were estimated, and BDI-IIs from every treatment session were included for the intervention group. Time was coded as 0 for baseline and as number of weeks from baseline for all subsequent measures. To control for differences in treatment duration the time frame was made comparable between groups by including only measures up to 7 weeks after baseline (the planned time frame for completing the intervention) for the intervention group in the main analysis. For the secondary measures and for analysis including the 6 months follow-up data on the BDI-II, repeated measures linear mixed-models analysis was performed with occasion (baseline, post-test, 6 months follow-up) as the repeated factor. This procedure was deemed acceptable since linear regression analyses did not find treatment duration to be a significant predictor of symptom change during the treatment phase of the intervention group for any of the secondary measures, $\beta = -.16 – .28$, $t = -0.91 – 1.58$, $P = .12 - .92$. Scores on the last BDI-II from participants completing 5 or more weeks of treatment but missing formal post-test data ($n = 8$) were included in the analysis, since this was considered to give a more accurate estimate of change over time.”

12a-i) Imputation techniques to deal with attrition / missing values

“Since mixed-models analysis can handle incomplete data, no procedure for imputation of missing data was utilised in the analysis [84].”

12b) CONSORT: Methods for additional analyses, such as subgroup analyses and adjusted analyses

“For completers, covariance analyses (ANCOVA) were performed with post symptom scores as the dependent variable and pre-intervention symptom scores and treatment duration as covariates.” “For all reported intention-to-treat and modified intention-to-treat analyses baseline anxiety symptoms (BAI) and age were included as covariates, with the exception that baseline BAI-scores were not included as covariate in the analysis of BAI.”

RESULTS

13a) CONSORT: For each group, the numbers of participants who were randomly assigned, received intended treatment, and were analysed for the primary outcome

“Number of participants who randomly assigned, received the intended treatment and were included in the primary analysis is shown in the consort flow diagram (Figure 2). “Of the 52 participants in the intervention group 31 (60 %) adhered to the treatment program, in that they completed MoodGYM and attended at least 7 sessions.”

13b) CONSORT: For each group, losses and exclusions after randomisation, together with reasons

Attrition after randomization and reasons for drop-out/non-adherence is shown in Figure 2 (consort flow diagram).

13b-i) Attrition diagram

Number of participants discontinuing the intervention is shown in the consort flow diagram. Other use parameters are described in the text. “Of the 52 participants in the intervention group 31 (60 %) adhered to the treatment program, in that they completed MoodGYM and attended at least 7 sessions. Overall, the sample starting treatment (n = 50) completed on average 3.8 of the 5 modules, attended a mean of 7.2 sessions, with average session duration of 27.7 minutes. The average number of weeks in treatment was 11.3. Total time spent by therapists ranged from 70 to 506 minutes (M = 242.1, SD = 96.6).” “Of the 50 participants starting treatment 86 % (n = 43) completed 2 or more modules, indicating that they may have completed sufficient of the treatment program to generate beneficial outcomes.”

14a) CONSORT: Dates defining the periods of recruitment and follow-up

“Participants (n=106) were recruited between October 2010 and October 2012.” The collection of 6-months follow-up data was completed in June 2013.

14a-i) Indicate if critical “secular events” fell into the study period

None.

14b) CONSORT: Why the trial ended or was stopped (early)

The trial ended when a sufficient number of participants were recruited.

15) CONSORT: A table showing baseline demographic and clinical characteristics for each group

Yes, Table 1 shows demographic and clinical variables for both groups at baseline, Table 2 shows pre-treatment scores on all outcome measures.

15-i) Report demographics associated with digital divide issues

Age, gender, education, employment status and Internet use frequency is reported in Table 1.

16a) CONSORT: For each group, number of participants (denominator) included in each analysis and whether the analysis was by original assigned groups

16-i) Report multiple “denominators” and provide definitions

Number of participants informed of the project, consented, and completed the intervention are presented in the consort flow diagram. A more detailed account of use for the intervention group is reported in the text for the intervention group (see 13b-i). Effect sizes are reported separately for intention-to-treat analyses and completer analyses.

16-ii) Primary analysis should be intent-to-treat

“Results on the BDI-II and BAI were analysed using intention-to-treat (ITT) analyses where participants are analysed in the group they were randomised to, irrespective of treatment adherence. Due to missing data at pre-test for the remaining secondary measures (3 % missing on the HADS, and 17 % and 19% missing on SWLS and EQ-5D, respectively), modified intention-to-treat analysis was performed including all participants completing the measures at least once.” Secondary analyses compared completers.

17a) CONSORT: For each primary and secondary outcome, results for each group, and the estimated effect size and its precision (such as 95% confidence interval)

Results for each group and estimated effect sizes are presented for both primary and secondary outcomes.

17a-i) Presentation of process outcomes such as metrics of use and intensity of use

We report average number of modules, average number of support sessions, average session duration and total time spent by the therapist during treatment (range, mean and standard deviation). We also report the proportion of participating all 5 modules and 2 modules (indicating that they may have completed sufficient of the treatment program to generate beneficial outcomes).

17b) CONSORT: For binary outcomes, presentation of both absolute and relative effect sizes is recommended

No binary outcomes.

18) CONSORT: Results of any other analyses performed, including subgroup analyses and adjusted analyses, distinguishing pre-specified from exploratory

Results of pre-specified completer analysis and analysis of clinically significant change (based on scores of the BDI-II) are reported. One exploratory analysis was included after being requested during the review process: “An exploratory linear regression analysis showed that symptom change in the intervention group was not significantly predicted by the total time spent by the therapists for any outcome measure, β = -.12 - .26, t = -0.81 – 1.52, P = .14 - .96.”

18-i) Subgroup analysis of comparing only users

The completer and exploratory analyses are clearly indicated.

19) CONSORT: All important harms or unintended effects in each group

One participant found that working with the program led to an increase in rumination and a worsening of depressive symptoms. Due to this the participant wished to discontinue the intervention (reported in the consort flow diagram). The participant discontinued the intervention, was encouraged to seek specialised mental health treatment and were offered help to obtain a referral.

19-i) Include privacy breaches, technical problems

Two of the e-mail reminders disclosed the presence of depressive symptoms and low level of self-esteem, respectively. According to the approval from the Regional Committee for Research Ethics in Northern Norway this e-mail content should have been encrypted. When this error was uncovered half way through the trial, the content was rephrased to exclude any information that could be regarded as sensitive. The possibility for privacy breaches related to this was reported to the ethics committee who approved the continuation of the trial and the usage of the data.

19-ii) Include qualitative feedback from participants or observations from staff/researchers

Result from qualitative interviews are reported in separate papers. The participants´ “frequently characterisation of the program as “too young”” is suggested as a possible explanation for the more moderate ratings of the web-program compared to the intervention as a whole.

DISCUSSION

20) CONSORT: Trial limitations, addressing sources of potential bias, imprecision, multiplicity of analyses

20-i) Typical limitations in ehealth trials

The lack of allocation concealment and blinding, and the role of the first and second authors as therapists in the trial introduced a risk of bias that may have inflated the treatment effects. Unfortunately, resource constraints prevented the use of independent therapists. The use of self-report measures rather than therapist assessments does to some degree alleviate this problem. Biased outcome assessments were further prevented by ensuring that a research assistant without knowledge of the participants' condition assignment collected post-test data. The sole reliance on self-report is a limitation in itself. Independent pre- and post-assessments by a clinician blinded to condition allocation would have been preferable and would have strengthened the results." "The multiplicity of outcomes increases the risk of Type I errors. However, the main findings of the present trial would still be significant when employing the Bonferroni correction. This indicates the robustness of the findings."

21) CONSORT: Generalisability (external validity, applicability) of the trial findings

21-i) Generalizability to other populations
"Although the heterogeneity of the sample and the recruitment from primary care is a strength, the generalizability of the results is uncertain since the sample was a self-selected group. Based on the notifications by the GPs when informing a patient of the study, the estimated uptake (meeting up for screening) in this study was 39 % (128 of 325 who received information), which is slightly above the median uptake for computerized CBT [54]. Considering the extra barriers imposed by the research activities this uptake rate is relatively high and indicates the possible acceptability of this treatment among depressed primary care patients. It also strengthens the generalizability of the results, by indicating that the self-selected group may be representative of a considerable proportion of the targeted group of primary care patients." "Further research is needed to investigate whether the intervention is truly acceptable for the wider group of primary care patients."

21-ii) Discuss if there were elements in the RCT that would be different in a routine application setting
"Since we for practical reasons have chosen to use psychologists for this first evaluation, further research is needed to determine if the present intervention would be as effective and acceptable in regular clinical practice, when delivered by GPs or other primary care therapists." "Further research is also needed to investigate whether the intervention is considered feasible and acceptable by general practitioners or other primary care therapists."

22) CONSORT: Interpretation consistent with results, balancing benefits and harms, and considering other relevant evidence

22-i) Restate study questions and summarize the answers suggested by the data, starting with primary outcomes and process outcomes (use)
"The results of the present study indicate that a guided self-help intervention combining the MoodGYM program with face-to-face therapist support can be effective in reducing depressive symptoms for a sample of mildly to moderately depressed individuals recruited from primary care. The intervention also had significant positive effects on symptoms of anxious worry and participants experienced significant improvements in global satisfaction with life, and, though non-significant, a tendency towards improved self-reported health-state. At 6-months follow-up positive gains in terms of reduction of depressive and anxious symptoms were largely maintained, while improvements in life satisfaction were partly maintained. The rate of non-adherence (40 %) was moderate and the evaluations of the treatment as a whole were predominately positive."

22-ii) Highlight unanswered new questions, suggest future research

"Since we for practical reasons have chosen to use psychologists for this first evaluation, further research is needed to determine if the present intervention would be as effective and acceptable in regular clinical practice, when delivered by GPs or other primary care therapists. It may also be noted that the present intervention was more time-intensive than the majority of other guided self-help interventions. However, since the role of the clinician was mainly supportive and facilitative and the main therapeutic input was delivered through a standardized treatment package, the intervention was regarded conceptually as a guided self-help intervention [31]. There has generally been found similar effects for low- and high-intensity guided internet-based psychotherapy [19]. Further research should investigate if the present intervention with more limited therapist support could yield similar effects."
"The intervention was designed to be suitable for implementation in primary health care, and could have a potential for use in a stepped-care approach. However, further research is necessary to determine whether it is equally effective when delivered in regular primary health care and whether the inclusion of booster session could further improve symptom maintenance. Further research is also needed to investigate whether the intervention is truly acceptable for the wider group of primary care patients and whether it is considered feasible and acceptable by general practitioners or other primary care therapists."

Other information

23) CONSORT: Registration number and name of trial registry
Trial registration: Australian New Zealand Clinical Trials Registry number: ACTRN12610000257066; https://www.anzctr.org.au/trial_view.aspx?id=335255 (Archived by WebCite® at http://www.webcitation.org/6GR48iZH4)

24) CONSORT: Where the full trial protocol can be accessed, if available

The protocol is included as appendix.

25) CONSORT: Sources of funding and other support (such as supply of drugs), role of funders

This study was financially funded by the Research Council of Norway (196423/V50). MoodGYM was provided by the Australian National University."

X26-i) Comment on ethics committee approval
The research protocol was approved by the Regional Committee for Research Ethics in Northern Norway (2011/2163) and the Human Ethics Committee of the Australian National University (ANU)."

x26-ii) Outline informed consent procedures
"Patients were provided with general information about the treatment and the aim of the project and detailed information about the methods for handling issues of privacy and anonymity. They were informed that they could expect to commence treatment within 6 weeks of the initial contact. To participate, patients sent in an informed consent form providing contact details." Trial information is included as appendix.

X26-iii) Safety and security procedures
"The web-based program did not store any personally identifying information about users." "After each module participants received face-to-face support. Symptoms of depression were monitored at every consultation and changes in symptom levels were discussed." The therapist could also be reached by phone between sessions. "Participants who experienced significant worsening in symptoms and were considered in need of more extensive treatment throughout or after completing the trial were assisted in the process of referral to specialised mental health services."

X27-i) State the relation of the study team towards the system being evaluated
"KG is one of the authors of the MoodGYM intervention evaluated in the trial and work for The Australian National University who provide free access to the MoodGYM websites. ME and KW contributed in the process of translating MoodGYM into Norwegian. RSH, KL and NK have no financial or non-financial interests to declare in relation to this study."
